# Cultivation and Genomics Prove Long-Term Colonization of Donor’s Bifidobacteria in Recurrent *Clostridioides difficile* Patients Treated With Fecal Microbiota Transplantation

**DOI:** 10.3389/fmicb.2020.01663

**Published:** 2020-07-15

**Authors:** Hanne Jouhten, Aki Ronkainen, Juhani Aakko, Seppo Salminen, Eero Mattila, Perttu Arkkila, Reetta Satokari

**Affiliations:** ^1^Human Microbiome Research Program, Faculty of Medicine, University of Helsinki, Helsinki, Finland; ^2^Functional Foods Forum, Faculty of Medicine, University of Turku, Turku, Finland; ^3^Department of Infectious Diseases, Helsinki University Hospital, Helsinki, Finland; ^4^Department of Gastroenterology, Helsinki University Hospital, Helsinki, Finland

**Keywords:** *C. difficile*, stool transplantation, intestinal microbiota, strain tracking, whole genome sequencing, comparative genomics, therapeutic bacteria, next-generation probiotics

## Abstract

Fecal microbiota transplantation (FMT) is an effective treatment for recurrent *Clostridioides difficile* infection (rCDI) and it’s also considered for treating other indications. Metagenomic studies have indicated that commensal donor bacteria may colonize FMT recipients, but cultivation has not been employed to verify strain-level colonization. We combined molecular profiling of *Bifidobacterium* populations with cultivation, molecular typing, and whole genome sequencing (WGS) to isolate and identify strains that were transferred from donors to recipients. Several *Bifidobacterium* strains from two donors were recovered from 13 recipients during the 1-year follow-up period after FMT. The strain identities were confirmed by WGS and comparative genomics. Our results show that specific donor-derived bifidobacteria can colonize rCDI patients for at least 1 year, and thus FMT may have long-term consequences for the recipient‘s microbiota and health. Conceptually, we demonstrate that FMT trials combined with microbial profiling can be used as a platform for discovering and isolating commensal strains with proven colonization capacity for potential therapeutic use.

## Introduction

In fecal microbiota transplantation (FMT), feces from a healthy donor is transplanted into a recipient in order to re-establish a healthy or normally functioning gut microbiota and to correct microbiota dysbiosis associated with the recipient’s condition. FMT has been highly effective in treating recurrent *Clostridioides difficile* infection (rCDI) and it is increasingly employed and recommended treatment for the disease (Surawicz et al., 2013; Cammarota et al., 2017). Gut microbiota of rCDI patients has generally a lower bacterial diversity and different taxonomical composition when compared to healthy individuals (Chang et al., 2008; Seekatz and Young, 2014). In rCDI patients, a successful treatment with FMT leads to increased microbial diversity and modified composition (Fuentes et al., 2014; Seekatz et al., 2014). Currently, rCDI is the only indication for FMT in clinical practice (Cammarota et al., 2017, 2019), but the treatment has shown promising results in other conditions where gut microbiota plays a role, such as inflammatory bowel disease (IBD), metabolic syndrome, and eradication of antibiotic resistant bacteria (Browne and Kelly, 2017; De Groot et al., 2017; Laffin et al., 2017). Several studies have demonstrated that microbiota of an FMT-treated rCDI patient is very similar to that of the donor, and there have been efforts to identify the commonly colonizing taxa along with specific set of bacteria that might be crucial for the success of such treatment (Jalanka et al., 2016; Staley et al., 2016). In indications such as IBD, the identity of specific bacteria may be even more important as early reports indicate that success may depend on the microbial profile of the donor (Moayyedi et al., 2015; Browne and Kelly, 2017). Thus, the potential of FMT in modifying gut microbiota, as well as the specific colonization of donor-derived taxa, has gained considerable interest. Donor strains that are able to colonize recipients in long-term are of special interest, as they might show potential for being so-called next-generation probiotics, i.e., therapeutic bacteria for the treatment of diseases in which dysbiosis is considered to play a role.

One study investigated the fate of donor-derived bacterial strains by shotgun-metagenomics and single nucleotide variant (SNV) analysis to track several bacterial species in five FMT-treated metabolic syndrome patients (Li et al., 2016). Considerable variation was observed in the transfer and persistence of donor-derived bacteria. Some donor strains either replaced or co-existed with the recipient strains, while some donor bacteria were not detected in recipient metagenomes. Interestingly, the colonization patterns were not similar among the recipients as individuals seemed to adopt donor-derived bacteria in a different manner. However, several donor strains were still detectable in all five recipient metagenomes 84 days post-FMT. The varied colonization of donor-derived species was also reported in a study comparing post-FMT metagenomes of seven rCDI patients, also by using SNV analysis (Kumar et al., 2017). Several donor-derived strains were detected in all seven recipients from 3 to 6 months post-FMT and a few strains were detected in two recipient metagenomes even 2 years after FMT. Thus, it seems that at least some donor-derived bacteria may colonize FMT recipients relatively permanently. More recently, a study combining metagenomics, strain identification based on single copy phylogenetic markers, and machine learning model assessed the colonization of donor bacteria in FMT recipients (Smillie et al., 2018). A total of 125 donor-derived strains were detected in the recipient metagenomes after FMT with 58 of these strains being detectable over a month post-FMT. Interestingly, closely related bacterial strains were observed to transfer mostly as sets in which all the strains of a distinct species were colonized, depending on the recipient, either in unison or not at all. Although several studies have addressed the colonization of non-pathogenic commensal donor bacteria by metagenomics, none of them has actually recovered donor strains from the recipients as pure cultures that would allow a precise strain identification and possible further use of effectively colonizing strains.

In this study, our aim was to assess the long-term colonization of donor-derived bifidobacteria in FMT recipients by combining culture-independent and culture-dependent methods. The study subjects comprised two FMT donors and thirteen rCDI patients. Sampling was performed prior to FMT as well as at various time points during the 1-year follow-up period. First, we analyzed fecal bifidobacterial populations of the donors and recipients by a genus-specific molecular profiling technique to detect putatively donor-derived bifidobacteria in the post-FMT samples of the recipients. Next, we isolated bifidobacteria from the samples of donors and recipients, and screened the bifidobacterial isolates by using rep-PCR typing. Finally, we selected 65 isolates for whole genome sequencing and comparative genomic analysis to identify the donor-derived strains among the recipient isolates.

## Materials and Methods

### FMT Donors and Recipients and Fecal Samples

Fecal samples (*n* = 112) originate from two FMT donors (DX and DY) and their thirteen recipients (DX: PX1–7, DY: PY1–6). The samples are presented in Supplementary Table 1. The recipients were rCDI patients successfully treated with FMT. Subjects and sampling have been described in detail earlier (Jalanka et al., 2016). Recipients donated a fecal sample prior to FMT as well as at different time points covering the 1-year follow-up period (Supplementary Table 1). Feces were stored at −80°C until the analyses.

### DNA Extractions

DNA extraction for *Bifidobacterium*-specific PCR-DGGE profiling of fecal samples included mechanical cell-lysis step by repeated bead-beating method described earlier (Salonen et al., 2010). DNA was purified from proteins with ammonium acetate, precipitated with isopropanol, washed with ethanol, and suspended in TE buffer. Genomic DNA (gDNA) of isolates for partial 16S rRNA gene sequencing, rep-PCR, and WGS by MiSeq was extracted with the same method from liquid cultures. Bacteria were grown in liquid MRS medium (BD) supplemented with 0.5 gl^–1^ of L-cysteine (Sigma-Aldrich) at 37°C for 48 ± 4 h under anaerobic atmosphere (85%N_2_, 10%CO_2_, and 5%H_2_). Bacterial pellets were harvested by centrifugation and suspended in RBB buffer. For WGS by PacBio, gDNA was extracted from cultures in midpoint logarithmic growth with MagAttract kit (Qiagen) according to the manufacturer’s instructions, preceded by 2-h chemical cell-lysis with MetaPolyzyme enzyme mixture (Sigma-Aldrich). DNA concentrations were determined with NanoDrop^TM^ ND-1000 Spectrophotometer (Thermo Scientific) or Qubit^®^2.0 fluorometer with Qubit dsDNA HS Assay kit (Thermo Scientific) according to the manufacturer’s instructions.

### PCR-DGGE Profiling

Bifidobacterial profiles of 112 fecal samples were analyzed by genus-specific PCR and denaturing gradient gel electrophoresis (DGGE) as described earlier (Satokari et al., 2001). Briefly, 5pmol of *Bifidobacterium*-specific primers Bif164-forward and Bif662-GC-reverse (Satokari et al., 2001) were added in reaction mixture of 25 μl containing 12.5 μl KAPA2G Robust HotStart ReadyMix (Kapa Biosystems) or MyTaq HS Red Mix (Bioline) and 1–150 ng DNA extracted from a fecal sample. If this failed, above-described PCR was done from 16S amplicon mixture produced with primers Bif164-forward and Bif662-reverse (Satokari et al., 2001) and called nested. Thermal cycler program was: 95°C for 3 min; 35 cycles of 95°C for 15 s, 62°C for 15 s, and 72°C for 15 s; and 72°C for 5 min. Amplicons were separated in polyacrylamide gels (Satokari et al., 2001) and DNA was stained with SYBR Green (BioWhittaker).

Digitalized gel images were imported into Bionumerics software (version 6.6; Applied Maths) for band detection with normalization conducted by known reference amplicons. After band search and matching (1% band tolerance as implemented in Bionumerics), the results were checked visually and corrected manually when necessary. The profiles were put through cluster analysis with unweighted pair group method with arithmetic mean (UPGMA) for constructing dendrogram based on Pearson correlation similarity coefficient, as implemented in Bionumerics. Data matrices containing band presence/absence information were exported to R (version 3.5.0; The R Foundation for Statistical Computing) for further analysis of clustering and sample similarity. Dissimilarity matrix was computed with daisy function from R-package cluster using Gower’s distance. Multiple correspondence analysis was performed by MCA function in R-package FactoMineR, and R-package factoextra functions fviz_mca_ind and fviz_contrib were used for visualization of sample clustering and contribution of variables in MCA, respectively. Statistically significant groups contributing to the sample clustering were identified by PERMANOVA analysis in adonis function from R-package vegan (Oksanen et al., 2018) using Bray-Curtis dissimilarities and 999 permutations.

### Isolation of Bifidobacteria and Tentative Identification

A subset of fecal samples used for PCR-DGGE profiling was selected for cultivation (*n* = 30; Supplementary Table 1). The subset included samples from the recipients whose profiles had indicated the presence of putative donor-derived bifidobacteria at different time points post-FMT. For each selected recipient, the sample at the latest post-FMT time point with putative donor-derived bifidobacteria was cultivated in order to isolate strains with long-term colonization capacity as well as one to three samples at earlier time points for strain comparison, including the pre-FMT sample if available. The subset included one sample from both donors whose profiles had been stable during the 1-year follow-up.

Cultivation was performed as described earlier (Quartieri et al., 2016) with slight modifications. Briefly, fecal samples were weighed and suspended 1:10 (w/v) in phosphate-buffered peptone water (Sigma-Aldrich) supplemented with 0.5 gl^–1^ of L-cysteine (Sigma-Aldrich). Next, 10-fold dilutions up to 10^–7^ were spread onto solid MRS medium (BD) supplemented with 0.5 gl^–1^ of L-cysteine (Sigma-Aldrich) and 0.05 gl^–1^ of mupirocin (Sigma-Aldrich) and grown at 37°C for 48 ± 4 h under anaerobic atmosphere. Several colonies of each type were picked from the lowest dilutions yielding single colonies. 163 isolates from purified cultures were examined for colony morphology, Gram reaction, and cell morphology. 10 isolates were discarded as they did not meet the criteria for bifidobacteria (Gram positive rods of various morphologies), and the remaining 153 isolates were subjected to taxonomic identification.

Taxonomic identification was done for isolates (*n* = 153) by sequencing a fragment of 16S rRNA gene. PCR mixtures of 25 μl contained 3.0 mM MgCl_2_, 0.2 mM dNTPs, 0.4 μM universal primers DegL, and pD (Turner et al., 1999) 1.0 U AmpliTaq Gold^®^ DNA polymerase (Applied Biosystems by Life Technologies), and 50–100 ng gDNA. Thermal cycler program was: 96°C for 2 min; 30 cycles of 96°C for 30 s, 56°C for 45 s, and 72°C for 1 min; 72°C for 5 min. Amplicons were Sanger sequenced in the Institute of Biotechnology (IB) at University of Helsinki (Helsinki, Finland) (UH) according to the institute’s protocols. The .ab1 trace files were processed with Staden Package software components pregap4 (version 1.6) and gap4 (version 4.11.2) (Bonfield et al., 1995). The resulting sequences were used as queries to search matches from the NCBI 16S ribosomal RNA nucleotide database by basic local alignment search tools (BLAST) megaBLAST algorithm (Morgulis et al., 2008) to assign species-level taxonomic identities. 6 isolates were discarded as they were not representatives of the genus *Bifidobacterium*, and the remaining 147 isolates were subjected to rep-PCR typing.

### Rep-PCR Typing

The isolates confirmed as *Bifidobacterium* sp. (*n* = 147) were typed by repetitive extragenic palindromic PCR (rep-PCR) (Jarocki et al., 2016). Briefly, the gDNAs were amplified with the BOXA1R primer (Jarocki et al., 2016). PCR mixtures of 20 μl contained 3.0 mM MgCl_2_, 0.2 mM dNTPs, 1.0 μM primer, 1.0 U AmpliTaq Gold^®^ DNA-polymerase (Applied Biosystems by Life Technologies), and 50 ng gDNA. Thermal cycler program was: 94°C for 4 min; 35 cycles 94°C for 1 min, 40°C for 1 min, 72°C for 2 min; 72°C for 10 min. The amplicons were electrophoresed in 1.4% agarose gel in TBE buffer at 120 V for 75 min for visualization.

### Whole Genome Sequencing

A subset of bifidobacterial isolates (*n* = 65; Supplementary Table 2) was selected for whole genome sequencing (WGS). The subset included donor isolates from all the different rep-PCR fingerprint types in order to verify tentative species-level identification as well as to observe within-species variation as indicated by the fingerprints. As the aim was to assess long-term colonization by donor strains, the subset included all but one recipient isolates that were considered donor-like by rep-PCR typing and were recovered from the time point of 4 months or beyond. The subset was supplemented with several similar and dissimilar recipient isolates of these species from different time points to bring about resolution in the whole genome analyses.

gDNA of 65 isolates were sequenced by Illumina MiSeq from Nextera XT genomic libraries (Illumina, Inc.) in the Institute for Molecular Medicine (FIMM) or in IB at UH with manufacturer protocols. The quality of sequences was checked with FastQC quality control tool (Andrews, 2010) and trimmed with Trimmomatic (Bolger et al., 2014) to remove adapters and cut low quality ends. The trimmed sequences were assembled into contigs with Spades (v3.13.0) assembly pipeline for paired sequences with k-mer lengths 21, 33, 55, 77, 99, and 127 (Nurk et al., 2013). In addition, two of the genomes were also sequenced with PacBio RSII (Pacific Biosciences of California, Inc.) using DNA/Polymerase binding kit P6 and assembled with HGA3 (SMRTportal 2.3.0) followed by polishing of the assemblies with MiSeq reads by Pilon-software (v1.23) (Walker et al., 2014) in the IB at UH. The quality of assemblies was analyzed by QUAST online-tool^1^ (Gurevich et al., 2013). In identifying the closest matches from the NCBI RefSeq genome database^2^, all the donor strain assemblies were converted into single-line FASTA files that were used as queries in BLAST search (Altschul et al., 1990).

### Comparative Genomics

Phylogenetic relationships of all the isolates (*n* = 65) and NCBI reference genomes (closest BLAST hits, listed in Supplementary Table 5) were studied with a phylogenomic approach by using command line tools of Anvi’o workflow (v5.5)^3^ (Eren et al., 2015) and FastTree (v2.1.10) (Price et al., 2010). FastTree implements an approximately-maximum-likelihood based approach, and it was used with default options, including 1,000 bootstraps. Briefly, after running the default HMM profiles, concatenated amino acid sequences of 49 single-copy genes of ribosomal proteins were aligned as implemented in the Anvi’o pipeline. Phylogenomic tree was constructed from the alignment by FastTree (v2.1.10) and visualized with FigTree (v1.4.4) (Rambaut, 2010). For whole genome SNP calling and construction of phylogenetic trees, we used CSIPhylogeny online-tool (v1.4)^4^ (Kaas et al., 2014). The two donor strain PacBio assemblies (DX_pv5PacBio and DX_pv32PacBio) were used as references against which the spades-assembled draft genomes of *B. longum* and *B. pseudocatenulatum* isolates were aligned with default parameters. A similar analysis was done for *B. adolescentis* isolates with the draft genome of DX_pv1 serving as reference. Trees were visualized with FigTree (v1.4.4) (Rambaut, 2010). Finally, the relationships of all the isolates were estimated by pangenomic analysis. The distribution of gene clusters across the genomes was estimated and visualized with command line tools of Anvi’o workflow (v5.5) for microbial pangenomics^5^. In the pipeline, blastp was chosen for search, MCL (Enright et al., 2002) for clustering, and muscle (Edgar, 2004) for alignment. Predicted proteins were functionally categorized based on their clusters of orthologous groups (COGs) (Galperin et al., 2015) and visualization of pangenome was drawn based on the presence/absence of gene clusters.

### Ethical Considerations

The study was approved by the Ethics Committee of Hospital District of Helsinki and Uusimaa Finland (DnroHUS124/13/03/01/11). The fecal donors and rCDI patients provided written informed consent to take part in the study.

### Data Availability

The whole genome sequencing data supporting the findings of this study have been deposited in European Nucleotide Archive (ENA) under study accession number: PRJEB35833. All other types of data generated or analyzed during this study are included in this article and its Supplementary Information.

## Results

### *Bifidobacterium*-Specific 16S rRNA Gene Profiles Suggest Transfer of Donor Bifidobacteria to FMT Recipients

Bifidobacterial profiles of the donors were both distinct and stable over time (Supplementary Figure 1). The profile of donor DY consisted of two bands that were in the same positions as those obtained from the donor’s *B. longum* and *B. pseudocatenulatum* isolates. The profile of donor DX was more complex, consisting of four or five bands during the 1-year follow-up period with a small change toward the end. The bands matched with those resulting from the donor’s *B. adolescentis*, *B. longum*, and *B. pseudocatenulatum* isolates.

**FIGURE 1:**
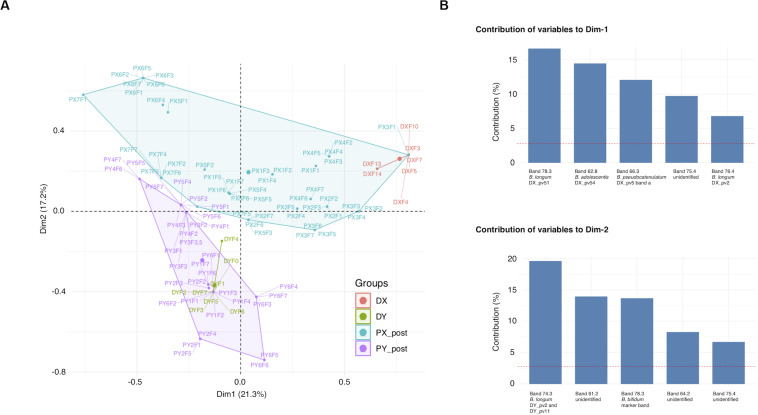
Clustering of *Bifidobacterium*-specific PCR-DGGE profiles obtained from fecal samples of FMT donors and their recipients at different time points. **(A)** MCA plot of the recipient post-FMT samples and donor samples. **(B)** The five band positions contributing the most to each dimension of MCA. DGGE, denaturing gradient gel electrophoresis; MCA, multiple correspondence analysis; DX and DY, FMT donors; PX1-7 and PY1-6, FMT recipients of DX and DY, respectively. Fecal sample time points for recipients and DY: F0, pre-FMT (the time of donation for DY); F1–F7, 3 days, 2 weeks, 1, 2, 4, 8, and 12 months post-FMT, respectively; Time points for DX: F3, the time of donation; F4, F5, F7, F10, F13, and F14, 2 weeks, 1, 2, 4, 8, and 12 months post-FMT, respectively.

Most of the recipient pre-FMT samples differed from all the other samples and clustered separately in the UPGMA cluster analysis, whereas many of the post-FMT samples grouped with the profiles of their respective donor (Supplementary Figure 1). Clustering of samples to the groups X or Y (samples of DX and DY and their respective recipients PX and PY) explained 20% (*p* = 0.001) of the post-FMT sample separation in MCA, whereas clustering to the groups D or P (donor or recipient) explained 7% of the separation (*p* = 0.001) (Figure 1A). The top five variables explaining the clustering of post-FMT samples in MCA included several bands matching the positions of those derived from the donor isolates, including all three analyzed *B. longum* donor isolates (Figure 1B). Thus, donor-recipient pairing influenced the clustering of the bifidobacterial PCR-DGGE profiles substantially, and the appearance of bands corresponding to the donor *B. longum* isolates contributed to the clustering the most.

### Donor-Like Bifidobacteria Were Isolated From FMT Recipients

**FIGURE 2:**
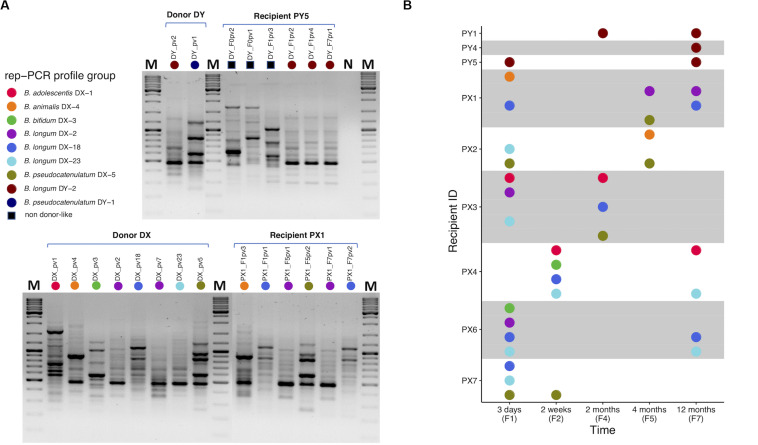
Rep-PCR typing of bifidobacteria isolated from fecal samples of FMT donors and recipients. **(A)** Representative gel pictures presenting rep-PCR fingerprint profiles of bifidobacterial isolates from donors DY and DX and their respective recipients PY5 and PX1 at different time points: PY5 has unique profiles pre-FMT, but also DY-like profiles post-FMT (upper gel). PX1 has DX-like profiles post-FMT (lower gel). **(B)** A visual summary of donor-like rep-PCR fingerprint profiles observed among each recipient’s isolates during the 1-year follow-up after FMT. Each colored dot refers to a distinct type of rep-PCR fingerprint profile observed among the donor isolates. A profile was indexed based on the first isolate to have that profile. For instance, purple dot representing profile *B. longum* DX-2 was indexed according to the donor isolate *B. longum* DX_pv2 in which the profile was encountered for the first time. If such profile was observed in a recipient isolate, the isolate was considered donor-like and received the same color. For instance, recipient isolate *B. longum* PX1_F7pv1 was observed to have *B. longum* DX-2 profile and received the purple color. Thus, all the donor and donor-like isolates with the same profile were considered representatives of a same rep-PCR profile group and putatively being of the same origin. All the profiles observed among recipient isolates but not in donor isolates were referred collectively with a black square **(A)**. PY1 and PY4-5, FMT recipients of DY; PX1-4 and PX6-7, FMT recipients of DX. The isolate code includes reference to the sample from which it was isolated, see Supplementary Table 2. M, GeneRuler^TM^ DNA Ladder Mix; N, negative PCR control.

Based on the results obtained from PCR-DGGE profiling, we chose one fecal sample from each of the donors and 28 from selected recipients (*n* = 30; Supplementary Table 1) for selective cultivation of bifidobacteria. Cultivation was successful from both donor samples and most of the recipient samples (23 out of 28) (Supplementary Table 3). The five negative recipient samples included two pre-FMT samples. A total of 153 bacterial isolates from the cultures were subjected to taxonomic identification by partial 16S rRNA gene sequencing with 147 isolates confirmed as *Bifidobacterium* spp. Altogether eight different species were represented among the isolates (Supplementary Table 2). Recipient pre-FMT samples were scarce in bifidobacteria: only one yielded isolates and they belonged to species that were not recovered from either of the donors (Supplementary Table 2) while the other cultivated pre-FMT samples were negative for bifidobacteria (Supplementary Table 3). The bifidobacterial isolates of same species exhibited similar phenotypic properties (colony and cell morphology; Supplementary Table 2). The overall variety of different species in the cultivated samples reflected the results obtained from PCR-DGGE profiling (Supplementary Table 3). Next, we subjected the isolates to rep-PCR typing in which 19 different fingerprint profiles were identified (Supplementary Table 2). Nine distinct rep-PCR fingerprint profiles were observed among the donor isolates revealing within-species variation and thus allowing resolution below species level (Figure 2A).

Two species of bifidobacteria were isolated from the donor DY and all the isolates within the same species had a similar rep-PCR fingerprint: *B. longum* (DY-2) and *B. pseudocatenulatum* (DY-1) (Figure 2A). Comparison of the donor and recipient isolates revealed that all three DY recipients carried DY-2-like *B. longum* 1 year after FMT (Figure 2B). However, no donor-like isolates of *B. pseudocatenulatum* were recovered from any of the DY recipients (Figure 2B). All the recipients carried also bifidobacterial species that were not isolated from the donor (Supplementary Table 2).

Donor DX had five bifidobacterial species and seven different rep-PCR fingerprints: *B. adolescentis* (DX-1), *B. animalis* (DX-4), *B. bifidum* (DX-3), *B. longum* (DX-2, DX-18, DX-23), and *B. pseudocatenulatum* (DX-5) (Figure 2A). All six recipients of DX had donor-like isolates after FMT (Figure 2B). Some recipients carried also unique strains of these species and one recipient had a species not isolated from the donor (Supplementary Table 2). The most prevalent donor-like isolates recovered from the recipients at the end of the 1-year follow-up period were strains of *B. longum* (DX-2, DX-18, or DX-23; Figure 2B). Donor-like *B. adolescentis* (DX-1) was also detected in one recipient 1 year post-FMT. Donor-like *B. pseudocatenulatum* (DX-5) was detected in multiple recipients up to 4 months post-FMT.

### Whole Genome Sequencing Verifies the Same Origin of Donor and Recipient Isolates

We subjected 65 bifidobacterial isolates (19 from the donors and 46 from the recipients; Supplementary Table 2) to WGS to assess the similarity of isolates by three different approaches: phylogenomics, phylogenetics, and pangenomics. The genome size, G+C content, and predicted gene count of all the isolates are presented in Supplementary Table 4. The assembly statistics of all the donor isolates along with the closest taxonomic matches obtained from the NCBI RefSeq genome database are presented in Supplementary Tables 4, 5, respectively.

**FIGURE 3:**
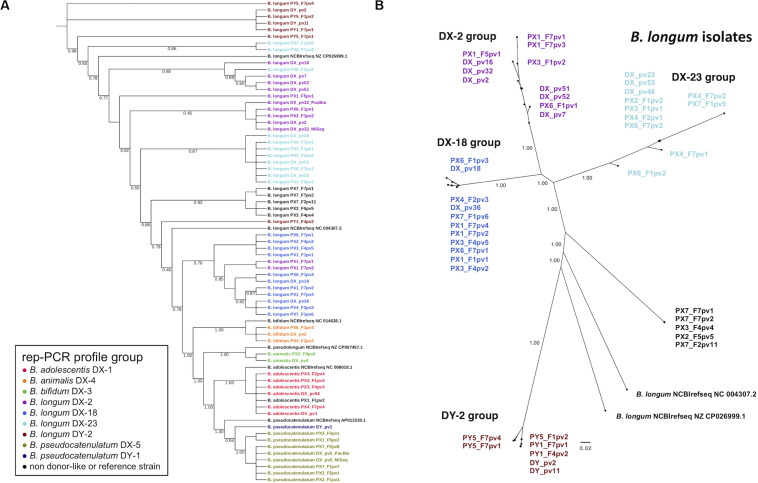
Phylogenetic trees of fecal bifidobacterial isolates from FMT donors and their recipients. **(A)** Phylogenomic tree based on concatenated amino acid sequences of 49 single copy genes of ribosomal proteins of all the isolates and NCBI reference genomes. Note that the tree is topological and branch distance is therefore unindicative of phylogenetic distance. **(B)** Phylogenetic tree of *B. longum* isolates based on whole genome SNP calling. The colors in isolate codes indicate an isolate’s rep-PCR fingerprint group. The branch support values represent proportions among 1,000 bootstraps. DX and DY, FMT donors; PX1-4 and PX6-7, FMT recipients of DX; PY1 and PY5, FMT recipients of DY. F1, F2, F4, F5, and F7, 3 days, 2 weeks, 2, 4, and 12 months post-FMT, respectively; pv1-54, isolate codes; DX_pv5_PacBio and DX_pv32_PacBio, two DX isolate genomes that were sequenced by PacBio in addition to MiSeq; REF, reference strains from the NCBI reference genome database (Supplementary Table 5).

To address the relatedness of the isolates we performed a phylogenomic analysis based on the alignment of concatenated amino acid sequences derived from 49 ribosomal protein genes for construction of phylogenetic tree. The isolates were clearly separated from the NCBI RefSeq genomes while displaying donor-strain-wise clustering (Figure 3A). Donor DX *B. longum* isolates representing the three different rep-PCR fingerprint groups separated into distinct phylogenetic clusters. Similarly, the two donor DY *B. longum* isolates with identical rep-PCR fingerprints clustered together. Several recipient isolates clustered together with the similar isolates from their respective donor.

To analyze the relatedness among *B. adolescentis*, *B. longum*, and *B. pseudocatenulatum* isolates from the donors and recipients more closely, the genomes were subjected to whole genome SNP calling to construct phylogenomic trees for each of the species. Phylogenomic groups were in accordance with the rep-PCR fingerprinting results: DX *B. longum* isolates and the similar recipient isolates were distributed into corresponding three groups (DX-2, DX-18, and DX-23 group) (Figure 3B). Likewise, the DY *B. longum* isolate and similar recipient isolates comprised their own group (DY-2 group). A group of recipient *B. longum* isolates not sharing a similar rep-PCR profile with the donor isolates comprised a separate group. In the phylogenomic trees of *B. adolescentis* and *B. pseudocatenulatum*, the isolates grouped according to their rep-PCR fingerprints (Supplementary Figure 2). SNP calling found 2213940–2273604, 2055193–2656944, and 2059398–2475233 positions for comparison among the genomes of *B. adolescentis*, *B. longum*, and *B. pseudocatenulatum*, respectively, and thus, the coverage of SNP analysis was as high as 75–99% of the genome lengths. The number of SNPs that separated clearly different strains (NCBI RefSeq genomes and the isolates originating from different donors) was about 7,000–8,000, whereas some isolates had only a few different SNPs indicating very close relatedness (Supplementary Table 6).

The SNP analysis revealed that the isolates clustering most closely in the trees were separated only by few or few hundred SNPs (Supplementary Table 6). In the *B. longum* DX-23 group, the difference between the donor isolates (DX_pv23, DX_pv48, and DX_pv53) and the four isolates from different recipients was less than ten SNPs. One of these recipient isolates (PX6_F7pv2) was recovered from 1-year post-FMT sample. The *B. longum* DX-18 group comprising two donor-isolates (DX_pv18 and DX_pv36) and eight recipient isolates had a corresponding difference up to 500 SNPs. Three of these recipient isolates originated from the 1-year post-FMT samples of two recipients (PX1_F7pv2 and PX1_F7pv4 separated by 165–187 SNPs from DX_pv36 and PX6_F7pv1 separated by 324 SNPs from DX_pv18). The donor strains of *B. longum* DY-2 group (DY_pv2 and DY_pv11) clustered together with three recipient isolates of which one originated from a 1-year post-FMT sample (PY1_F7pv1 separated from the donor isolates by some 200 SNPs; Supplementary Table 6).

**FIGURE 4:**
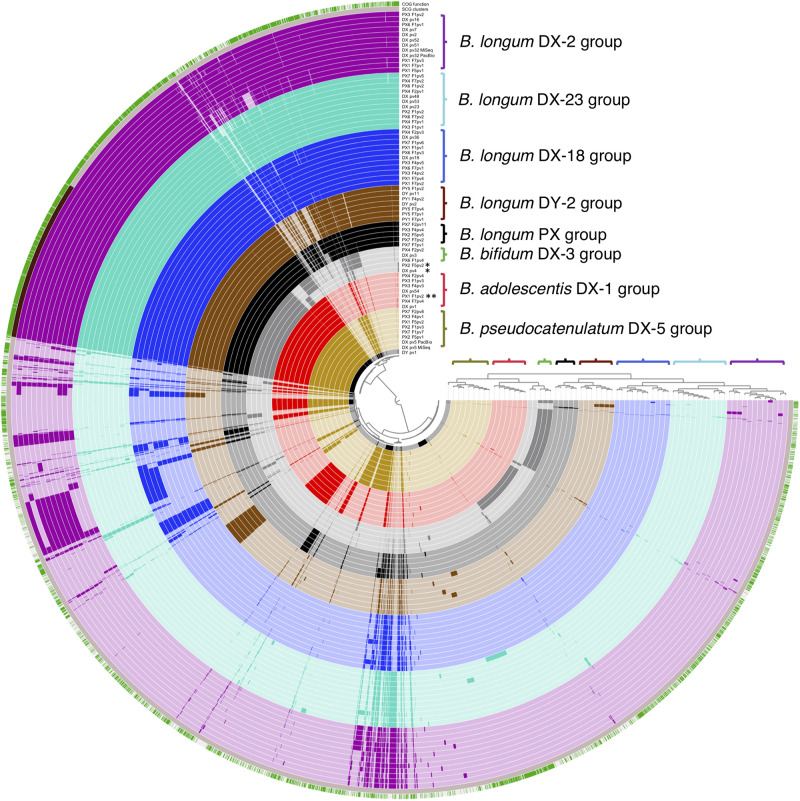
Visual comparison of genomic content of all the 65 sequenced bifidobacterial isolates from FMT donors and their recipients. Colored brackets with group labels refer to the rep-PCR fingerprint groups. The upper layer presents genomic content with known and unknown clusters of orthologous groups (COGs) in green and white, respectively. The second layer presents core genome as single copy gene (SCG) functions in dark brown. DX and DY, FMT donors; PX1-4 and PX6-7, FMT recipients of DX; PY1 and PY5, FMT recipients of DY; F1, F2, F4, F5, and F7, 3 days, 2 weeks, 2, 4, and 12 months post-FMT, respectively; pv1-54, isolate codes; DX_pv5_PacBio and DX_pv32_PacBio, two DX isolate genomes that were sequenced by PacBio in addition to MiSeq; *, *B. animalis* isolate; **, *B. adolescentis* isolate not belonging to the DX-1 group.

We also conducted a pangenome analysis to view the differences in the distribution of gene clusters among all the isolates. The pipeline estimated that the genomes were 97–98.5% complete and revealed genome sizes, G+C content, and gene numbers as shown in Supplementary Table 4. An average gene cluster number per genome was 2024 (min. 1562, max. 2369). The shared part of genomes (belonging to the core genome of all the isolates) consisted of 800 gene clusters containing 540 single-copy core gene clusters coding mainly known clusters of orthologous groups (COGs) (Figure 4). The accessory genome varied between the genomes and contained more clusters with unknown COGs. Importantly, the pangenome analysis separated the genomes into groups that were in accordance with the rep-PCR fingerprint groups of the isolates as well as with the groups displayed by phylogenetic trees, and thus further confirmed the similarity of strains isolated from each of the donor and their corresponding recipients. In summary, all our analyses demonstrated that strains of donor groups *B. adolescentis* DX-1, *B. longum* DX-18, *B. longum* DX-23, and *B. longum* DY-2 were recovered from the recipients until a year after FMT and strains of donor group *B. pseudocatenulatum* DX-5 until 4 months post-FMT.

## Discussion

FMT is an effective treatment for rCDI and its capacity to modify recipient gut microbiota to donor-like configuration is well-established (Khoruts et al., 2010; Weingarden et al., 2015; Broecker et al., 2016; Fuentes and De Vos, 2016; Jalanka et al., 2016). However, the fate of specific donor strains in the gut microbiota of recipients is still unclear (Shankar et al., 2014; Broecker et al., 2016; Moss et al., 2017; Staley et al., 2017). Recent metagenomic studies and SNV mapping have addressed this question and indicated presence of donor strains in recipients for several months after FMT (Li et al., 2016; Kumar et al., 2017; Lee et al., 2017; Smillie et al., 2018). Recently, Drewes et al. (2019) assessed the presence of bacterial virulence factors up to 6 months after FMT in eleven pediatric rCDI patients and their respective donors by fecal cultures and quantitative PCR, and the results indicated durable transmission. However, the strain verification by WGS was done only in one patient-donor-pair. Another recent study revealed the transfer of extended-spectrum beta-lactamase (ESBL) producing *Escherichia coli* to two recipients from one fecal donor who had not been pre-screened for the pathogen (Defilipp et al., 2019). While these studies employed cultivation and WGS to demonstrate the transfer of donor bacteria to recipients, our study greatly extends the previous observations by involving more donor-recipient-pairs as well as having a longer follow-up period and a larger number of studied isolates. Our study is the first one to demonstrate the transfer of non-pathogenic commensal bacteria in several donor-recipient-pairs by cultivation and strain verification by WGS. Furthermore, we were able to demonstrate the long-term colonization of donor-derived strains in the FMT recipients as specific strains were detected in multiple recipients even up to 1 year post-FMT.

We targeted fecal bifidobacterial populations in FMT donors and recipients by genus-specific PCR-DGGE profiling at different time points during a 1-year period. The method allows a rapid assessment of changes occurring in a specific group of bacteria and it is well suited for bifidobacterial population dynamics at an individual level (Satokari et al., 2001). The donor profiles were stable over time, which is in accordance with previous observations on bifidobacterial populations in healthy Western adults (Satokari et al., 2001; Rajilic-Stojanovic et al., 2012). Also, the diversity of bifidobacteria in the donors resembled that observed for healthy adults by using the same PCR-DGGE method (Satokari et al., 2001). Many of the recipient pre-FMT samples were depleted on bifidobacteria as they were either negative in the genus-specific PCR (7 out of 12 samples) or yielded an amplification product only by using the more sensitive nested PCR (3 out of 7 samples) (Supplementary Table 3). This finding is in line with previous observations that CDI patients have typically lower abundance of bifidobacteria as compared to healthy individuals (Amrane et al., 2019). In contrast, bifidobacterial PCR was successful with most recipient post-FMT samples (80 out of 85), and the DGGE profiles indicated the presence of multiple strains. In our previous study assessing total microbiota changes in the same cohort of rCDI patients, we observed over a 5-fold increase in the signal level from bifidobacteria in the phylogenetic microarray analysis (Jalanka et al., 2016). We revisited the microarray data and constituted that the relative abundance of bifidobacteria in the majority of our rCDI patients was below 0.8% before FMT, but increased to an average 4% post-FMT, whereas the donors Y and X had ∼2 and 8% relative abundance of bifidobacteria, respectively (data not shown). Taken together, both the qualitative analysis performed in this study and the previous semiquantitative results from the phylogenetic microarray profiling indicate that FMT drastically enriched bifidobacteria in the rCDI patients, in addition to the total microbiota re-establishment.

The recipient samples clustered together with the respective donor samples in the UPGMA analysis indicating a high similarity in the bifidobacterial population structure. We analyzed factors contributing to the grouping of post-FMT recipient profiles together with the donor profiles and found the band positions of donor *B. longum* strains to be major factors. As the bands in the recipient post-FMT profiles matched to those obtained from donor isolates, we found it plausible that they represent transferred bifidobacteria. Some recipient post-FMT profiles contained also unique community members, suggesting that FMT may also have fostered the recovery of endogenous bifidobacterial populations of the recipients. Previously, a mouse model with CDI and severe dysbiosis showed that administration of a bacterial cocktail of six species was able to trigger major shifts in the microbial community structure and to induce the recovery of endogenous microbiota (Lawley et al., 2012).

We cultivated bifidobacteria on selective MUP medium, which promotes bifidobacterial growth very well while also being selective for the genus (Quartieri et al., 2016). The samples for cultivation were selected based on the profiling of bifidobacterial populations with the aim of recovering donor-derived strains from the recipients. The vast majority of the isolates were identified as *Bifidobacterium* spp. by typical phenotypic traits such as cell morphology as well as by partial 16S rRNA gene sequencing. The isolates were subjected to molecular typing by rep-PCR, which expectedly allowed separation below the species level (Jarocki et al., 2016). Comparison of rep-PCR fingerprints from the donor and recipient isolates revealed that all the recipients carried some donor-like bifidobacterial strains after FMT. Importantly, some of the donor-like strains present in the recipient post-FMT samples could be isolated months or even a year after the treatment and the strain identities were confirmed by WGS analysis. It has been previously reported that stable colonization of probiotic *B. longum* AH1206 depends on the features of recipient gut microbiota as they define the ecological niches present in the environment (Maldonado-Gomez et al., 2016). Usually, allochthonous bifidobacteria introduced into the adult gut, such as orally administered probiotic strains, are typically lost after a few days or weeks without their continuous influx (Satokari et al., 2001; Mättö et al., 2006). On the other hand, *B. longum* has been documented to transmit vertically as certain strains in mother’s feces or breast milk have been detected in child’s fecal samples even up to 6 years after birth (Duranti et al., 2017; Oki et al., 2018). Thus, bifidobacteria seem capable of establishing themselves as permanent residents of the human gut, at least during the development of gut microbiota when an ecological niche is available. As rCDI patients usually have aberrant and less abundant gut microbiota, it might provide bifidobacteria with niches not necessarily present in healthy microbiota. Indeed, a recent mouse study demonstrated that antibiotic pre-treatment prior to FMT enhances specifically the colonization of bifidobacteria (Freitag et al., 2019). In this study, all the recipients were rCDI patients who had received multiple antibiotic treatments before FMT, which may have contributed to the observed colonization of bifidobacteria. Overall, rCDI patients have highly depleted microbiota with poor colonization resistance capacity and the likelihood of stable strain colonization is probably much higher than in healthy gut environment. However, we isolated and identified strains with proven long-term colonization capacity, but the capacity still needs to be addressed in other populations than rCDI patients and with the isolated strains available such further studies are feasible.

In order to verify the same origin of the isolated bifidobacterial strains from the donors and recipients, we subjected 65 isolates to WGS to compare their similarity by three different approaches including phylogenomic, phylogenetic, and pangenomic analysis. The isolate genome sizes, G+C contents, and predicted gene numbers were similar to those obtained for fecal bifidobacteria in previous studies (Duranti et al., 2017; Freitas and Hill, 2018). In the phylogenomic and phylogenetic analysis, the isolates clustered according to their rep-PCR fingerprints. We also analyzed the relatedness of donor and recipient strains by whole genome SNP calling with CSIPhylogeny tool, which is well suited for example for outbreak surveillance based on whole genome sequencing data (Kaas et al., 2014; Saltykova et al., 2018). The tool is particularly well suited for closely related strains as it provides more informative sites and detects more feasibly high-quality SNPs for comparison (Kaas et al., 2014). Here, we performed separate runs for *B. adolescentis*, *B. longum*, and *B. pseudocatenulatum* isolates. Based on the analysis, several *B. longum* strains of the donor DX and the *B. longum* strain of the donor DY were very close relatives with multiple DX and DY recipient isolates, respectively. Moreover, several isolates originating from different time points from the same recipient as well as isolates from different recipients who had received FMT from the same donor were found to be closely related. Similarly, *B. adolescentis* and *B. pseudocatenulatum* isolates from the DX recipients were highly similar to the corresponding DX isolates. While it is clear that isolates differing by only a few SNPs, such as all the donor strains and majority of recipient isolates in the DX-23 group, represent the same strain, we find it likely that also the isolates differing up to several hundred SNPs originate from the same strain. It should be noted that some of the strains were recovered from the recipients months or even a year after FMT and certainly genomic changes, such as those seen in the SNP analysis, could have accumulated during that time. Similarly, strains in a donor microbiota could have diverged into several lineages from a single ancestor strain, which could explain the SNP differences found among the strains isolated from the same donor. Naturally, sequencing errors may also contribute to the observed differences. The number of SNPs between the NCBI RefSeq genomes or isolates obtained from the different donors was ∼7000–8000, which shows that strains with distinct origin could be clearly indicated. Regarding the strain separation, there is no consensus defining the number of SNPs that could differentiate one strain from another, and the results are dependent on the species as well as on the quality of sequences (Hilliard et al., 2018; Saltykova et al., 2018). Our analysis was able to compare over two million sites from every isolate and covered 99–100% of the whole genomes of *B. adolescentis*, 75–96% of *B. longum*, and 82–99% of *B. pseudocatenulatum*, thus giving good coverage of the genomes and great confidence of the results. Furthermore, the pangenome analysis supported the grouping based on the other methods. Taken together, the comprehensive analysis of the bifidobacterial isolates confirmed the same origin of several donor and recipient strains and showed that specific strains transferred via FMT can persist in rCDI patients for long term.

Here we used our previous clinical study on the FMT treatment of rCDI patients (Jalanka et al., 2016) as a discovery platform for isolating and selecting bifidobacterial strains with proven capacity for long-term colonization, and confirmed by WGS that the strains acquired by the recipients originated from the donors. The most prominent colonizers belong to the species *B. longum* in both donor-recipient groups. All DY recipients carried similar *B. longum* strains 1 year after FMT, whereas DX recipients had three different persistent strains. One DX recipient had also acquired a donor-derived strain of *B. adolescentis* that was detectable 1 year post-FMT. Interestingly, a recent study combining both metagenomic and culturomic approaches found that CDI patients lack two bifidobacterial species, *B. adolescentis* and *B. longum* (Amrane et al., 2019). The observation is in line with our results and is encouraging regarding the potential use of these species in future bacteriotherapeutic applications. In this regard, bifidobacteria have already a long history as probiotics and many specific strains hold a “generally regarded as safe” status (GRAS) in the United States and are on the Qualitative Presumption of Safety (QPS) list in the European Union, making their use easy and straightforward both in probiotic foods and as therapeutic agents or supplements. In fact, strains of *Bifidobacterium* have already been included in a bacterial mix that was used to treat successfully two rCDI patients (Petrof et al., 2013).

We demonstrate that FMT trials accompanied by microbiota analysis can be used as discovery platforms to identify and isolate bacteria that can effectively colonize dysbiotic human gut and be used as novel probiotics for therapeutic purposes. The results obtained can guide the design of further studies focusing on bacteriotherapeutic cocktails based on *in-vitro*-cultivated bacteria. Concerning the isolated strains, the most obvious question to be addressed is the factors behind long-term colonization. On the bacterial side, mechanisms mediating adhesion to intestinal mucus and enterocytes as well as efficient nutrient harvest in the very competitive gut environment can contribute to successful colonization (Ventura et al., 2012). Our pangenomic analysis revealed that, unlike the core genome, the gene clusters unique to certain groups had no functional annotation. Previously, Bottacini et al. (2014) hypothesized that such unique parts of genomes may code for novel *Bifidobacterium*-specific molecules involved in host-bacterial interactions. While all our donor strains can be considered to be well adapted to the gut environment as they showed remarkably stable existence as part of bifidobacterial communities in the donors, there was a clear difference in their long-term colonization success across the recipients. We didn’t observe an “all-or-none” mode of colonization of donor bifidobacteria in the recipients, as was found for closely related strains by Smillie et al. (2018). Indeed, the successful colonization seems to depend on several factors including the bacterial species in question, possible competition or mutualism within the species as well as the resident microbiota, and the immune responses of the recipient, and thus, personalized approaches may also need to be considered to achieve stable gut colonization.

## Data Availability Statement

The datasets presented in this study can be found in online repositories. The names of the repository/repositories and accession number(s) can be found at: https://www.ebi.ac.uk/ena, PRJEB35833.

## Ethics Statement

The studies involving human participants were reviewed and approved by the Ethics Committee of Hospital District of Helsinki and Uusimaa Finland (DnroHUS124/13/03/01/11). The patients/participants provided their written informed consent to participate in this study.

## Author Contributions

RS designed and conceived the study. RS, EM, and PA carried out the underlying clinical study and collected the samples and clinical data. HJ, AR, and RS designed the experiments, interpreted the results, and wrote the manuscript. HJ and AR conducted the experiments and data analysis. JA and SS provided expertise and input for the DGGE analysis. AR and RS revised the manuscript according to the reviewers’ comments. All authors revised the manuscript for important intellectual content and accepted the final version.

## Conflict of Interest

The authors declare that the research was conducted in the absence of any commercial or financial relationships that could be construed as a potential conflict of interest.

## Footnotes

1 http://quast.sourceforge.net/quast

2 https://www.ncbi.nlm.nih.gov/refseq/

3 http://merenlab.org/2017/06/07/phylogenomics/

4 https://cge.cbs.dtu.dk/services/CSIPhylogeny/

5 http://merenlab.org/2016/11/08/pangenomics-v2/

## References

[B1] Altschul S. F. Gish W. Miller W. Myers E. W. Lipman D. J. (1990). Basic local alignment search tool. *J. Mol. Biol.* 215 403–410. 10.1016/S0022-2836(05)80360-2 2231712

[B2] Amrane S. Hocquart M. Afouda P. Kuete E. Pham T. P. Dione N. (2019). Metagenomic and culturomic analysis of gut microbiota dysbiosis during Clostridium difficile infection. *Sci. Rep.* 9:12807. 10.1038/s41598-019-49189-8 31488869 PMC6728329

[B3] Andrews S. (2010). *FastQC**: A Quality Control Tool for High Throughput Sequence Data.* Available online at: http://www.bioinformatics.babraham.ac.uk/projects/fastqc (accessed February 20, 2019).

[B4] Bolger A. M. Lohse M. Usadel B. (2014). Trimmomatic: a flexible trimmer for Illumina sequence data. *Bioinformatics* 30 2114–2120. 10.1093/bioinformatics/btu170 24695404 PMC4103590

[B5] Bonfield J. K. Smith K. Staden R. (1995). A new DNA sequence assembly program. *Nucleic Acids Res.* 23 4992–4999. 10.1093/nar/23.24.4992 8559656 PMC307504

[B6] Bottacini F. Ventura M. Van Sinderen D. O’connell Motherway M. (2014). Diversity, ecology and intestinal function of bifidobacteria. *Microb. Cell Fact.* 13(Suppl. 1):S4. 10.1186/1475-2859-13-S1-S4 25186128 PMC4155821

[B7] Broecker F. Klumpp J. Schuppler M. Russo G. Biedermann L. Hombach M. (2016). Long-term changes of bacterial and viral compositions in the intestine of a recovered Clostridium difficile patient after fecal microbiota transplantation. *Cold Spring Harb. Mol. Case Stud.* 2:a000448. 10.1101/mcs.a000448 27148577 PMC4849847

[B8] Browne A. S. Kelly C. R. (2017). Fecal transplant in inflammatory bowel disease. *Gastroenterol. Clin. North Am.* 46 825–837. 10.1016/j.gtc.2017.08.005 29173524

[B9] Cammarota G. Ianiro G. Kelly C. R. Mullish B. H. Allegretti J. R. Kassam Z. (2019). International consensus conference on stool banking for faecal microbiota transplantation in clinical practice. *Gut* 68 2111–2121. 10.1136/gutjnl-2019-319548 31563878 PMC6872442

[B10] Cammarota G. Ianiro G. Tilg H. Rajilic-Stojanovic M. Kump P. Satokari R. (2017). European consensus conference on faecal microbiota transplantation in clinical practice. *Gut* 66 569–580. 10.1136/gutjnl-2016-313017 28087657 PMC5529972

[B11] Chang J. Y. Antonopoulos D. A. Kalra A. Tonelli A. Khalife W. T. Schmidt T. M. (2008). Decreased diversity of the fecal microbiome in recurrent Clostridium difficile-associated diarrhea. *J. Infect. Dis.* 197 435–438. 10.1086/525047 18199029

[B12] De Groot P. F. Frissen M. N. De Clercq N. C. Nieuwdorp M. (2017). Fecal microbiota transplantation in metabolic syndrome: history, present and future. *Gut Microbes* 8 253–267. 10.1080/19490976.2017.1293224 28609252 PMC5479392

[B13] Defilipp Z. Bloom P. P. Torres Soto M. Mansour M. K. Sater M. R. A. Huntley M. H. (2019). Drug-resistant *E. coli* bacteremia transmitted by fecal microbiota transplant. *N. Engl. J. Med.* 381 2043–2050. 10.1056/NEJMoa1910437 31665575

[B14] Drewes J. L. Corona A. Sanchez U. Fan Y. Hourigan S. K. Weidner M. (2019). Transmission and clearance of potential procarcinogenic bacteria during fecal microbiota transplantation for recurrent *Clostridioides difficile*. *JCI Insight* 4:e130848. 10.1172/jci.insight.130848 31578306 PMC6795395

[B15] Duranti S. Lugli G. A. Mancabelli L. Armanini F. Turroni F. James K. (2017). Maternal inheritance of bifidobacterial communities and bifidophages in infants through vertical transmission. *Microbiome* 5:66. 10.1186/s40168-017-0282-6 28651630 PMC5485682

[B16] Edgar R. C. (2004). MUSCLE: multiple sequence alignment with high accuracy and high throughput. *Nucleic Acids Res.* 32 1792–1797. 10.1093/nar/gkh340 15034147 PMC390337

[B17] Enright A. J. Van Dongen S. Ouzounis C. A. (2002). An efficient algorithm for large-scale detection of protein families. *Nucleic Acids Res.* 30 1575–1584. 10.1093/nar/30.7.1575 11917018 PMC101833

[B18] Eren A. M. Esen O. C. Quince C. Vineis J. H. Morrison H. G. Sogin M. L. (2015). Anvi’o: an advanced analysis and visualization platform for ’omics data. *PeerJ* 3:e1319 10.7717/peerj.1319 PMC4614810 26500826

[B19] Freitag T. L. Hartikainen A. Jouhten H. Sahl C. Meri S. Anttila V. J. (2019). Minor effect of antibiotic pre-treatment on the engraftment of donor microbiota in fecal transplantation in mice. *Front. Microbiol.* 10:2685. 10.3389/fmicb.2019.02685 31824463 PMC6881239

[B20] Freitas A. C. Hill J. E. (2018). Bifidobacteria isolated from vaginal and gut microbiomes are indistinguishable by comparative genomics. *PLoS One* 13:e0196290. 10.1371/journal.pone.0196290 29684056 PMC5912743

[B21] Fuentes S. De Vos W. M. (2016). How to manipulate the microbiota: fecal microbiota transplantation. *Adv. Exp. Med. Biol.* 902 143–153. 10.1007/978-3-319-31248-4_10 27161356

[B22] Fuentes S. Van Nood E. Tims S. Heikamp-De Jong I. Ter Braak C. J. Keller J. J. (2014). Reset of a critically disturbed microbial ecosystem: faecal transplant in recurrent Clostridium difficile infection. *ISME J.* 8 1621–1633. 10.1038/ismej.2014.13 24577353 PMC4817604

[B23] Galperin M. Y. Makarova K. S. Wolf Y. I. Koonin E. V. (2015). Expanded microbial genome coverage and improved protein family annotation in the COG database. *Nucleic Acids Res.* 43 D261–D269. 10.1093/nar/gku1223 25428365 PMC4383993

[B24] Gurevich A. Saveliev V. Vyahhi N. Tesler G. (2013). QUAST: quality assessment tool for genome assemblies. *Bioinformatics* 29 1072–1075. 10.1093/bioinformatics/btt086 23422339 PMC3624806

[B25] Hilliard A. Leong D. O’callaghan A. Culligan E. P. Morgan C. A. Delappe N. (2018). Genomic characterization of Listeria monocytogenes isolates associated with clinical listeriosis and the food production environment in Ireland. *Genes* 9:171. 10.3390/genes9030171 29558450 PMC5867892

[B26] Jalanka J. Mattila E. Jouhten H. Hartman J. De Vos W. M. Arkkila P. (2016). Long-term effects on luminal and mucosal microbiota and commonly acquired taxa in faecal microbiota transplantation for recurrent *Clostridium difficile* infection. *BMC Med.* 14:155. 10.1186/s12916-016-0698-z 27724956 PMC5057499

[B27] Jarocki P. Podlesny M. Komon-Janczara E. Kucharska J. Glibowska A. Targonski Z. (2016). Comparison of various molecular methods for rapid differentiation of intestinal bifidobacteria at the species, subspecies and strain level. *BMC Microbiol.* 16:159. 10.1186/s12866-016-0779-3 27449060 PMC4957357

[B28] Kaas R. S. Leekitcharoenphon P. Aarestrup F. M. Lund O. (2014). Solving the problem of comparing whole bacterial genomes across different sequencing platforms. *PLoS One* 9:e104984 10.1371/journal.pone.0104984 PMC4128722 25110940

[B29] Khoruts A. Dicksved J. Jansson J. K. Sadowsky M. J. (2010). Changes in the composition of the human fecal microbiome after bacteriotherapy for recurrent Clostridium difficile-associated diarrhea. *J. Clin. Gastroenterol.* 44 354–360. 10.1097/MCG.0b013e3181c87e02 20048681

[B30] Kumar R. Yi N. Zhi D. Eipers P. Goldsmith K. T. Dixon P. (2017). Identification of donor microbe species that colonize and persist long term in the recipient after fecal transplant for recurrent Clostridium difficile. *NPJ Biofilms Microbiomes* 3:12 10.1038/s41522-017-0020-7 PMC5462795 28649413

[B31] Laffin M. Millan B. Madsen K. L. (2017). Fecal microbial transplantation as a therapeutic option in patients colonized with antibiotic resistant organisms. *Gut Microbes* 8 221–224. 10.1080/19490976.2016.1278105 28059612 PMC5479404

[B32] Lawley T. D. Clare S. Walker A. W. Stares M. D. Connor T. R. Raisen C. (2012). Targeted restoration of the intestinal microbiota with a simple, defined bacteriotherapy resolves relapsing Clostridium difficile disease in mice. *PLoS Pathog.* 8:e1002995 10.1371/journal.ppat.1002995 PMC3486913 23133377

[B33] Lee S. T. M. Kahn S. A. Delmont T. O. Shaiber A. Esen O. C. Hubert N. A. (2017). Tracking microbial colonization in fecal microbiota transplantation experiments via genome-resolved metagenomics. *Microbiome* 5:50. 10.1186/s40168-017-0270-x 28473000 PMC5418705

[B34] Li S. S. Zhu A. Benes V. Costea P. I. Hercog R. Hildebrand F. (2016). Durable coexistence of donor and recipient strains after fecal microbiota transplantation. *Science* 352 586–589. 10.1126/science.aad8852 27126044

[B35] Maldonado-Gomez M. X. Martinez I. Bottacini F. O’callaghan A. Ventura M. Van Sinderen D. (2016). Stable engraftment of Bifidobacterium longum AH1206 in the human gut depends on individualized features of the resident microbiome. *Cell Host Microbe* 20 515–526. 10.1016/j.chom.2016.09.001 27693307

[B36] Mättö J. Fondén R. Tolvanen T. Von Wright A. Vilpponen-Salmela T. Satokari R. (2006). Intestinal survival and persistence of probiotic Lactobacillus and Bifidobacterium strains administered in triple-strain yoghurt. *Int. Dairy J.* 16 1174–1180. 10.1016/j.idairyj.2005.10.007

[B37] Moayyedi P. Surette M. G. Kim P. T. Libertucci J. Wolfe M. Onischi C. (2015). Fecal microbiota transplantation induces remission in patients with active ulcerative colitis in a randomized controlled tria. *Gastroenterology* 149:l102-109.e106. 10.1053/j.gastro.2015.04.001 25857665

[B38] Morgulis A. Coulouris G. Raytselis Y. Madden T. L. Agarwala R. Schaffer A. A. (2008). Database indexing for production MegaBLAST searches. *Bioinformatics* 24 1757–1764. 10.1093/bioinformatics/btn322 18567917 PMC2696921

[B39] Moss E. L. Falconer S. B. Tkachenko E. Wang M. Systrom H. Mahabamunuge J. (2017). Long-term taxonomic and functional divergence from donor bacterial strains following fecal microbiota transplantation in immunocompromised patients. *PLoS One* 12:e0182585. 10.1371/journal.pone.0182585 28827811 PMC5565110

[B40] Nurk S. Bankevich A. Antipov D. Gurevich A. A. Korobeynikov A. Lapidus A. (2013). Assembling single-cell genomes and mini-metagenomes from chimeric MDA products. *J. Comput. Biol.* 20 714–737. 10.1089/cmb.2013.0084 24093227 PMC3791033

[B41] Oki K. Akiyama T. Matsuda K. Gawad A. Makino H. Ishikawa E. (2018). Long-term colonization exceeding six years from early infancy of *Bifidobacterium longum* subsp. longum in human gut. *BMC Microbiol.* 18:209. 10.1186/s12866-018-1358-6 30541439 PMC6292050

[B42] Oksanen J. Blanchet F. G. Friendly M. Kindt R. Legendre P. Mcglinn D. (2018). *vegan: Community Ecology Package. R package version 2.5-2.* Available online at: https://CRAN.R-project.org/package=vegan (accessed July 18, 2019).

[B43] Petrof E. O. Gloor G. B. Vanner S. J. Weese S. J. Carter D. Daigneault M. C. (2013). Stool substitute transplant therapy for the eradication of *Clostridium difficile* infection: ‘RePOOPulating’ the gut. *Microbiome* 1:3. 10.1186/2049-2618-1-3 24467987 PMC3869191

[B44] Price M. N. Dehal P. S. Arkin A. P. (2010). FastTree 2–approximately maximum-likelihood trees for large alignments. *PLoS One* 5:e9490. 10.1371/journal.pone.0009490 20224823 PMC2835736

[B45] Quartieri A. Simone M. Gozzoli C. Popovic M. D’auria G. Amaretti A. (2016). Comparison of culture-dependent and independent approaches to characterize fecal bifidobacteria and lactobacilli. *Anaerobe* 38 130–137. 10.1016/j.anaerobe.2015.10.006 26481833

[B46] Rajilic-Stojanovic M. Heilig H. G. Tims S. Zoetendal E. G. De Vos W. M. (2012). Long-term monitoring of the human intestinal microbiota composition. *Environ. Microbiol.* 10.1111/1462-2920.12023 [Online ahead of print] 23286720

[B47] Rambaut A. (2010). *FigTree v1.3.1.* Edinburgh: University of Edinburgh.

[B48] Salonen A. Nikkila J. Jalanka-Tuovinen J. Immonen O. Rajilic-Stojanovic M. Kekkonen R. A. (2010). Comparative analysis of fecal DNA extraction methods with phylogenetic microarray: effective recovery of bacterial and archaeal DNA using mechanical cell lysis. *J. Microbiol. Methods* 81 127–134. 10.1016/j.mimet.2010.02.007 20171997

[B49] Saltykova A. Wuyts V. Mattheus W. Bertrand S. Roosens N. H. C. Marchal K. (2018). Comparison of SNP-based subtyping workflows for bacterial isolates using WGS data, applied to *Salmonella enterica* serotype *Typhimurium* and serotype 1,4,[5],12:i. *PLoS One* 13:e0192504. 10.1371/journal.pone.0192504 29408896 PMC5800660

[B50] Satokari R. M. Vaughan E. E. Akkermans A. D. Saarela M. De Vos W. M. (2001). Bifidobacterial diversity in human feces detected by genus-specific PCR and denaturing gradient gel electrophoresis. *Appl. Environ. Microbiol.* 67 504–513. 10.1128/AEM.67.2.504-513.2001 11157210 PMC92614

[B51] Seekatz A. M. Aas J. Gessert C. E. Rubin T. A. Saman D. M. Bakken J. S. (2014). Recovery of the gut microbiome following fecal microbiota transplantation. *mBio* 5:e00893-14. 10.1128/mBio.00893-14 24939885 PMC4068257

[B52] Seekatz A. M. Young V. B. (2014). Clostridium difficile and the microbiota. *J. Clin. Invest.* 124 4182–4189. 10.1172/JCI72336 25036699 PMC4191019

[B53] Shankar V. Hamilton M. J. Khoruts A. Kilburn A. Unno T. Paliy O. (2014). Species and genus level resolution analysis of gut microbiota in Clostridium difficile patients following fecal microbiota transplantation. *Microbiome* 2:13. 10.1186/2049-2618-2-13 24855561 PMC4030581

[B54] Smillie C. S. Sauk J. Gevers D. Friedman J. Sung J. Youngster I. (2018). Strain tracking reveals the determinants of bacterial engraftment in the human gut following fecal microbiota transplantation. *Cell Host Microbe* 23:229-240.e225. 10.1016/j.chom.2018.01.003 29447696 PMC8318347

[B55] Staley C. Kelly C. R. Brandt L. J. Khoruts A. Sadowsky M. J. (2016). Complete microbiota engraftment is not essential for recovery from recurrent Clostridium difficile infection following fecal microbiota transplantation. *mBio* 7:e01965-16. 10.1128/mBio.01965-16 27999162 PMC5181777

[B56] Staley C. Vaughn B. P. Graiziger C. T. Singroy S. Hamilton M. J. Yao D. (2017). Community dynamics drive punctuated engraftment of the fecal microbiome following transplantation using freeze-dried, encapsulated fecal microbiota. *Gut Microbes* 8 276–288. 10.1080/19490976.2017.1299310 28282270 PMC5479395

[B57] Surawicz C. M. Brandt L. J. Binion D. G. Ananthakrishnan A. N. Curry S. R. Gilligan P. H. (2013). Guidelines for diagnosis, treatment, and prevention of Clostridium difficile infections. *Am. J. Gastroenterol.* 108 478–498. 10.1038/ajg.2013.4 23439232

[B58] Turner S. Pryer K. M. Miao V. P. Palmer J. D. (1999). Investigating deep phylogenetic relationships among cyanobacteria and plastids by small subunit rRNA sequence analysis. *J. Eukaryot. Microbiol.* 46 327–338. 10.1111/j.1550-7408.1999.tb04612.x 10461381

[B59] Ventura M. Turroni F. Motherway M. O. Macsharry J. Van Sinderen D. (2012). Host-microbe interactions that facilitate gut colonization by commensal bifidobacteria. *Trends Microbiol.* 20 467–476. 10.1016/j.tim.2012.07.002 22902802

[B60] Walker B. J. Abeel T. Shea T. Priest M. Abouelliel A. Sakthikumar S. (2014). Pilon: an integrated tool for comprehensive microbial variant detection and genome assembly improvement. *PLoS One* 9:e112963. 10.1371/journal.pone.0112963 25409509 PMC4237348

[B61] Weingarden A. Gonzalez A. Vazquez-Baeza Y. Weiss S. Humphry G. Berg-Lyons D. (2015). Dynamic changes in short- and long-term bacterial composition following fecal microbiota transplantation for recurrent Clostridium difficile infection. *Microbiome* 3:10. 10.1186/s40168-015-0070-0 25825673 PMC4378022

