# Supplementary material for: Cultivation and Genomics Prove Long-Term Colonization of Donor’s Bifidobacteria in Recurrent Clostridioides difficile Patients Treated With Fecal Microbiota Transplantation
Source: Front Microbiol. 2020 Jul 15;11:1663. doi: 10.3389/fmicb.2020.01663 (PMC7373762; doi:10.3389/fmicb.2020.01663)

Supplementary Material

| **Supplementary Table 2. Summary of bifidobacterial isolates.** | | | | | |  | |  |  |  |
| --- | --- | --- | --- | --- | --- | --- | --- | --- | --- | --- |
|  |  |  |  | |  |  | |  |  |  |
|  | **Subject ID** | **Sample^1^** | **Isolate^2^** | | **Colony morphology^3^** | **Cell morphology^4^** | | **16S rDNA gene sequencing** | **rep-PCR type** | **Other sample isolates with similar rep-PCR type^2^** |
|  | DX | F3 | **DX_pv1** | | grey, flat | polymorphic | | B. adolescentis | DX-1 | DX_pv8, DX_pv9, DX_pv15, DX_pv25, DX_pv26, DX_pv29, DX_pv30, DX_pv31, DX_pv33, **DX_pv54** |
|  | DX | F3 | **DX_pv2** | | grey, slimy | long, curved | | B. longum | DX-2 | **DX_pv7**, **DX_pv16**, DX_pv17, **DX_pv32**, DX_pv46, DX_pv47, **DX_pv51**, **DX_pv52** |
|  | DX | F3 | **DX_pv3** | | white, edged | long, curved | | B. bifidum | DX-3 | - |
|  | DX | F3 | **DX_pv4** | | white, edged | short, thick | | B. animalis | DX-4 | DX_pv10, DX_pv11, DX_pv13, DX_pv14, DX_pv19, DX_pv20, DX_pv21, DX_pv22, DX_pv34, DX_pv35 |
|  | DX | F3 | **DX_pv5** | | white, slimy | long, thick | | B. pseudocatenulatum | DX-5 | DX_pv27, DX_pv28 |
|  | DX | F3 | **DX_pv18** | | grey, slimy | long, curved | | B. longum | DX-18 | **DX_pv36**, DX_pv44 |
|  | DX | F3 | **DX_pv23** | | grey, slimy | long, curved | | B. longum | DX-23 | DX_pv24, DX_pv37, DX_pv38, DX_pv39, DX_pv40, DX_pv41, DX_pv42, DX_pv43, DX_pv45, **DX_pv48**, DX_pv49, DX_pv50, **DX_pv53** |
|  | PX1 | F1 | **PX1_F1pv1** | | grey, slimy | long, curved | | B. longum | DX-18-like | - |
|  | PX1 | F1 | **PX1_F1pv2** | | grey, flat | polymorphic | | B. adolescentis | PX1-F1-1 | - |
|  | PX1 | F1 | PX1_F1pv3 | | white, edged | long, curved | | B. animalis | DX-4-like | - |
|  | PX1 | F5 | **PX1_F5pv1** | | grey, slimy | long, curved | | B. longum | DX-2-like | PX1_F5pv3, PX1_F5pv6 |
|  | PX1 | F5 | **PX1_F5pv2** | | white, slimy | long, thick | | B. pseudocatenulatum | DX-5-like | PX1_F5pv4, PX1_F5pv5 |
|  | PX1 | F7 | **PX1_F7pv1** | | grey, slimy | long, curved | | B. longum | DX-2-like | **PX1_F7pv3** |
|  | PX1 | F7 | **PX1_F7pv2** | | grey, slimy | long, curved | | B. longum | DX-18-like | **PX1_F7pv4**, PX1_F7pv5 |
|  | PX2 | F1 | **PX2_F1pv2** | | grey, slimy | long, curved | | B. longum | DX-23-like | PX2_F1pv4 |
|  | PX2 | F1 | **PX2_F1pv3** | | white, slimy | long, thick | | B. pseudocatenulatum | DX-5-like | PX2_F1pv5 |
|  | PX2 | F5 | **PX2_F5pv1** | | white, slimy | long, thick | | B. pseudocatenulatum | DX-5-like | - |
|  | PX2 | F5 | **PX2_F5pv2** | | white, edged | short, thick | | B. animalis | DX-4-like | - |
|  | PX2 | F5 | **PX2_F5pv5** | | grey, slimy | long, curved | | B. longum | PX2-F5-5* | - |
|  | PX3 | F1 | **PX3_F1pv1** | | white, rough | long, curved | | B. longum | DX-23-like | - |
|  | PX3 | F1 | **PX3_F1pv2** | | grey, slimy | long, curved | | B. longum | DX-2-like | - |
|  | PX3 | F1 | **PX3_F1pv3** | | grey, flat | polymorphic | | Bifidobacterium sp. | DX-1-like | PX3_F1pv4 |
|  | PX3 | F4 | **PX3_F4pv1** | | white, slimy | long, thick | | B. pseudocatenulatum | DX-5-like | - |
|  | PX3 | F4 | **PX3_F4pv2** | | grey, slimy | long, curved | | B. longum | DX-18-like | **PX3_F4pv5** |
|  | PX3 | F4 | **PX3_F4pv3** | | grey, flat | polymorphic | | B. adolescentis | DX-1-like | - |
|  | PX3 | F4 | **PX3_F4pv4** | | grey, slimy | long, curved | | B. longum | PX3-F4-4* | - |
|  | PX4 | F2 | **PX4_F2pv1** | | grey, slimy | long, curved | | B. longum | DX-23-like | - |
|  | PX4 | F2 | **PX4_F2pv2** | | white, edged | long, curved | | B. bifidum | DX-3-like | - |
|  | PX4 | F2 | **PX4_F2pv3** | | grey, slimy | long, curved | | B. longum | DX-18-like | PX4_F2pv5 |
|  | PX4 | F2 | **PX4_F2pv4** | | grey, flat | polymorphic | | B. adolescentis | DX-1-like | - |
|  | PX4 | F7 | **PX4_F7pv1** | | grey, slimy | long, curved | | B. longum | DX-23-like | **PX4_F7pv2**, PX4_F7pv3, PX4_F7pv5 |
|  | PX4 | F7 | **PX4_F7pv4** | | grey, flat | polymorphic | | B. adolescentis | DX-1-like | - |
|  | PX6 | F1 | **PX6_F1pv1** | | grey, slimy | long, curved | | B. longum | DX-2-like | - |
|  | PX6 | F1 | **PX6_F1pv2** | | grey, slimy | long, curved | | B. longum | DX-23-like | - |
|  | PX6 | F1 | **PX6_F1pv3** | | grey, slimy | long, curved | | B. longum | DX-18-like | - |
|  | PX6 | F1 | **PX6_F1pv4** | | white, edged | long, curved | | B. bifidum | DX-3-like | - |
|  | PX6 | F7 | **PX6_F7pv1** | | grey, slimy | long, curved | | B. longum | DX-18-like | - |
|  | PX6 | F7 | **PX6_F7pv2** | | grey, slimy | long, curved | | B. longum | DX-23-like | - |
|  | PX6 | F7 | PX6_F7pv3 | | white, edged | short, thick | | B. breve | PX6-F7-3 | - |
|  | PX7 | F1 | **PX7_F1pv5** | | grey, slimy | long, curved | | B. longum | DX-23-like | - |
|  | PX7 | F1 | **PX7_F1pv6** | | grey, slimy | long, curved | | B. longum | DX-18-like | - |
|  | PX7 | F1 | **PX7_F1pv7** | | white, slimy | long, thick | | B. pseudocatenulatum | DX-5-like | - |
|  | PX7 | F1 | PX7_F1pv8 | | grey, flat | polymorphic | | B. adolescentis | PX7-F1-8 | - |
|  | PX7 | F2 | PX7_F2pv7 | | white, slimy | long, thick | | B. pseudocatenulatum | DX-5-like | **PX7_F2pv8**, PX7_F2pv9, PX7_F2pv10 |
|  | PX7 | F2 | **PX7_F2pv11** | | grey, slimy | long, curved | | B. longum | PX7-F2-11* | - |
|  | PX7 | F2 | PX7_F2pv12 | | grey, flat | polymorphic | | B. adolescentis | PX7-F2-12 | PX7_F2pv13 |
|  | PX7 | F7 | **PX7_F7pv1** | | grey, slimy | long, curved | | B. longum | PX7-F2-11-like | **PX7_F7pv2**, PX7_F7pv3, PX7_F7pv4, PX7_F7pv5 |
|  | DY | F2 | **DY_pv1** | | white, slimy | long, thick | | B. pseudocatenulatum | DY-1 | DY_pv3, DY_pv4, DY_pv5, DY_pv7, DY_pv10, DY_pv12, DY_pv13 |
|  | DY | F2 | **DY_pv2** | | white, edged | long, curved | | B. longum | DY-2 | DY_pv6, **DY_pv11** |
|  | PY1 | F1 | PY1_F1pv1 | | white, edged | short, thick | | B. animalis | PY1-F1-1 | - |
|  | PY1 | F4 | **PY1_F4pv2** | | white, edged | long, curved | | B. longum | DY-2-like | - |
|  | PY1 | F4 | PY1_F4pv3 | | white, edged | short, thick | | B. animalis | PY1-F1-1-like | - |
|  | PY1 | F7 | **PY1_F7pv1** | | white, edged | long, curved | | B. longum | DY-2-like | PY1_F7pv2 |
|  | PY4 | F1 | PY4_F1pv2 | | white, edged | short, thick | | B. animalis | PY4-F1-2 | PY4_F1pv3 |
|  | PY4 | F3 | PY4_F3pv2 | | white, edged | short, thick | | B. animalis | PY4-F1-2-like | - |
|  | PY4 | F7 | PY4_F7pv6 | | white, edged | long, curved | | B. longum | DY-2-like | - |
|  | PY4 | F7 | PY4_F7pv7 | | white, edged | short, thick | | B. animalis | PY4-F1-2-like | - |
|  | PY5 | F0 | PY5_F0pv1 | | grey | short, thick | | B. pseudolongum | PY5-F0-1 | - |
|  | PY5 | F0 | PY5_F0pv2 | | white, rough | short, thick | | B. dentium | PY5-F0-2 | PY5_F0pv3, PY5_F0pv4 |
|  | PY5 | F1 | **PY5_F1pv2** | | white, edged | long, curved | | B. longum | DY-2-like | PY5_F1pv4 |
|  | PY5 | F1 | PY5_F1pv3 | | white, edged | long, curved | | B. bifidum | PY5-F1-3 | - |
|  | PY5 | F7 | **PY5_F7pv1** | | white, edged | long, curved | | B. longum | DY-2-like | PY5_F7pv2, PY5_F7pv3, **PY5_F7pv4** |
|  | ^1^See Supplementary Table 1 for sample designations. | | | | |  | |  |  |  |
|  | ^2^Isolates in bold were later subjected to whole genome sequencing. | | | | |  | |  |  |  |
|  | ^3^Colony morphology on MRS medium. | | | |  |  | |  |  |  |
|  | ^4^Cell morphology in Gram stain. | | | |  |  | |  |  |  |
|  | ^*^Recipient rep-PCR types PX2-F5-5, PX3-F4-4, and PX7-F2-11 were similar to each other. This type was not present among their donor's isolates. | | | | | | | | |  |
|  |  |  | |  |  | |  |  |  |  |

| **Supplementary Table 3. Combined results from PCR-DGGE profiling and cultivation.** | | | | | |
| --- | --- | --- | --- | --- | --- |
|  |  |  |  |  |  |
|  | **Subject ID** | **Sample^1^** | **PCR-DGGE (No. of bands)** | **Cultivation^2^** | |
|  |  |  |  | **No. of species** | **No. of rep-PCR types** |
|  | DX | F3 | 6 | 5 | 7 |
|  | DX | F4 | 6 | - | - |
|  | DX | F5 | 6 | - | - |
|  | DX | F7 | 6 | - | - |
|  | DX | F10 | 6 | - | - |
|  | DX | F13 | 5 | - | - |
|  | DX | F14 | 5 | - | - |
|  | PX1 | F0 | 3 | no sample available | |
|  | PX1 | F1 | 4 | 3 | 3 |
|  | PX1 | F2 | 4 | - | - |
|  | PX1 | F3 | 4 | - | - |
|  | PX1 | F4 | 4 | - | - |
|  | PX1 | F5 | 3 | 2 | 2 |
|  | PX1 | F6 | 3 | - | - |
|  | PX1 | F7 | 3 | 1 | 2 |
|  | PX2 | F0 | 2 (normal and nested PCR) | 0 | 0 |
|  | PX2 | F1 | 4 | 2 | 2 |
|  | PX2 | F2 | 4 | - | - |
|  | PX2 | F3 | 4 | - | - |
|  | PX2 | F4 | 3 | - | - |
|  | PX2 | F5 | 3 | 3 | 3 |
|  | PX2 | F6 | 1 | - | - |
|  | PX2 | F7 | 1 | 0 | 0 |
|  | PX3 | F0 | no PCR product | no sample available | |
|  | PX3 | F1 | 6 | 2 | 3 |
|  | PX3 | F2 | 4 | 0 | 0 |
|  | PX3 | F3 | 4 | - | - |
|  | PX3 | F4 | 4 | 3 | 4 |
|  | PX3 | F5 | 3 | - | - |
|  | PX3 | F6 | 3 | - | - |
|  | PX3 | F7 | 3 | 0 | 0 |
|  | PX4 | F0 | 3 | no sample available | |
|  | PX4 | F1 | no sample available | no sample available | |
|  | PX4 | F2 | 3 | 3 | 4 |
|  | PX4 | F3 | 3 | - | - |
|  | PX4 | F4 | 3 | - | - |
|  | PX4 | F5 | 3 | - | - |
|  | PX4 | F6 | 2 | - | - |
|  | PX4 | F7 | 2 | 2 | 2 |
|  | PX5 | F0 | no sample available | no sample available | |
|  | PX5 | F1 | 4 | - | - |
|  | PX5 | F2 | 3 | - | - |
|  | PX5 | F3 | 4 | - | - |
|  | PX5 | F4 | 3 | - | - |
|  | PX5 | F5 | 3 | - | - |
|  | PX5 | F6 | 3 | - | - |
|  | PX5 | F7 | no PCR product | - | - |
|  | PX6 | F0 | no PCR product | no sample available | |
|  | PX6 | F1 | 5 | 2 | 4 |
|  | PX6 | F2 | 5 | - | - |
|  | PX6 | F3 | 5 | - | - |
|  | PX6 | F4 | 3 | - | - |
|  | PX6 | F5 | 5 | - | - |
|  | PX6 | F6 | 5 | - | - |
|  | PX6 | F7 | 5 | 2 | 3 |
|  | PX7 | F0 | 1 | 0 | 0 |
|  | PX7 | F1 | 5 | 3 | 4 |
|  | PX7 | F2 | 3 | 3 | 3 |
|  | PX7 | F3 | 3 | - | - |
|  | PX7 | F4 | 3 | - | - |
|  | PX7 | F5 | 2 | - | - |
|  | PX7 | F6 | 3 | - | - |
|  | PX7 | F7 | 3 | 1 | 1 |
|  | DY | F0 | 2 | - | - |
|  | DY | F1 | 2 | - | - |
|  | DY | F2 | 2 | 2 | 2 |
|  | DY | F3 | 2 | - | - |
|  | DY | F4 | 2 | - | - |
|  | DY | F5 | 2 | - | - |
|  | DY | F6 | 2 | - | - |
|  | DY | F7 | 2 | - | - |
|  | PY1 | F0 | no PCR product | no sample available | |
|  | PY1 | F1 | 2 | 1 | 1 |
|  | PY1 | F2 | 2 | - | - |
|  | PY1 | F3 | 2 | - | - |
|  | PY1 | F4 | 2 | 2 | 2 |
|  | PY1 | F5 | no PCR product | - | - |
|  | PY1 | F6 | 1 | - | - |
|  | PY1 | F7 | 1 | 1 | 1 |
|  | PY2 | F0 | 1 (nested PCR) | - | - |
|  | PY2 | F1 | 3 | - | - |
|  | PY2 | F2 | 2 | - | - |
|  | PY2 | F3 | 2 | - | - |
|  | PY2 | F4 | 3 | - | - |
|  | PY2 | F5 | 3 | - | - |
|  | PY2 | F6 | no sample available | no sample available | |
|  | PY2 | F7 | no sample available | no sample available | |
|  | PY3 | F0 | no PCR product | - | - |
|  | PY3 | F1 | 2 | - | - |
|  | PY3 | F2 | 2 | - | - |
|  | PY3 | F3 | 2 | - | - |
|  | PY3 | F3.5 | 2 | - | - |
|  | PY3 | F4 | no sample available | no sample available | |
|  | PY3 | F5 | no sample available | no sample available | |
|  | PY3 | F6 | no sample available | no sample available | |
|  | PY3 | F7 | no sample available | no sample available | |
|  | PY4 | F0 | 1 or 2 (two nested PCRs) | no sample available | |
|  | PY4 | F1 | 2 | 1 | 1 |
|  | PY4 | F2 | 2 | - | - |
|  | PY4 | F3 | 1 | 1 | 1 |
|  | PY4 | F4 | no PCR product | - | - |
|  | PY4 | F5 | no PCR product | - | - |
|  | PY4 | F6 | 2 | - | - |
|  | PY4 | F7 | 2 | 2 | 2 |
|  | PY5 | F0 | 2 | 2 | 2 |
|  | PY5 | F1 | 1 | 2 | 2 |
|  | PY5 | F2 | 1 | - | - |
|  | PY5 | F3 | no PCR product | - | - |
|  | PY5 | F4 | 1 | - | - |
|  | PY5 | F5 | 1 | - | - |
|  | PY5 | F6 | 1 | - | - |
|  | PY5 | F7 | 1 | 1 | 1 |
|  | PY6 | F0 | 2 (nested PCR) | - | - |
|  | PY6 | F1 | 2 | - | - |
|  | PY6 | F2 | 2 | - | - |
|  | PY6 | F3 | 3 | - | - |
|  | PY6 | F4 | 3 | - | - |
|  | PY6 | F5 | 4 | - | - |
|  | PY6 | F6 | 4 | - | - |
|  | PY6 | F7 | 3 | - | - |
|  | ^1^See Supplementary Table 1 for sample designations. | | |  |  |
|  | ^2^See Supplementary Table 2 for exact species identities and rep-PCR types. | | | |  |

| **Supplementary Table 4. Summary of genome assembly statistics from Quast quality assessment tool.** | | | | | |  |  |  |  |  |  |
| --- | --- | --- | --- | --- | --- | --- | --- | --- | --- | --- | --- |
|  |  |  |  |  |  |  |  |  |  |  |  |
|  | **Isolate** | **No. of**  **contigs** | **Largest**  **contig** | **Total**  **length** | **GC (%)** | **N50** | **N70** | **L50** | **L75** | **No. of N's per 100 kbp** | **No. of predicted genes GeneMarkS** |
|  | B. adolescentis DX_pv1 | 18 | 1337422 | 2219354 | 59,56 | 1337422 | 173973 | 1 | 3 | 0 | 1812 |
|  | B. adolescentis DX_pv54 | 16 | 1052189 | 2217587 | 59,57 | 253524 | 151909 | 2 | 5 | 0 | 1809 |
|  | B. animalis DX_pv4 | 15 | 531323 | 1919712 | 60,47 | 481401 | 114334 | 2 | 5 | 0 | 1561 |
|  | B. bifidum DX_pv3 | 11 | 617906 | 2247245 | 62,66 | 415771 | 315666 | 3 | 4 | 0 | 1914 |
|  | B. longum DX_pv2 | 71 | 230418 | 2640184 | 59,92 | 86974 | 45695 | 10 | 20 | 0 | 2341 |
|  | B. longum DX_pv7 | 76 | 230418 | 2623541 | 59,93 | 87644 | 51531 | 11 | 20 | 0 | 2300 |
|  | B. longum DX_pv16 | 110 | 269951 | 2492702 | 60,19 | 88353 | 38843 | 9 | 21 | 0 | 2216 |
|  | B. longum DX_pv18 | 98 | 125472 | 2568039 | 60,28 | 68150 | 37229 | 15 | 28 | 0 | 2243 |
|  | B. longum DX_pv23 | 57 | 260095 | 2396895 | 60,12 | 118909 | 61648 | 7 | 14 | 0 | 2030 |
|  | B. longum DX_pv32_PacBio | 5 | 2514508 | 2725031 | 60,08 | 2514508 | 2514508 | 1 | 1 | 0 | 2353 |
|  | B. longum DX_pv32_MiSeq | 93 | 220110 | 2661228 | 59,99 | 53518 | 32134 | 14 | 30 | 0 | 2351 |
|  | B. longum DX_pv36 | 102 | 124715 | 2751026 | 60,04 | 71744 | 37230 | 15 | 29 | 0 | 2460 |
|  | B. longum DX_pv48 | 56 | 242331 | 2393727 | 60,13 | 111558 | 64458 | 8 | 15 | 0 | 2028 |
|  | B. longum DX_pv51 | 66 | 230418 | 2617069 | 59,94 | 88091 | 45695 | 11 | 21 | 0 | 2280 |
|  | B. longum DX_pv52 | 85 | 198946 | 2613075 | 59,95 | 82258 | 37311 | 12 | 23 | 0 | 2288 |
|  | B. longum DX_pv53 | 59 | 232497 | 2398936 | 60,13 | 102403 | 64455 | 8 | 16 | 0 | 2028 |
|  | B. pseudocatenulatum DX_pv5_PacBio | 1 | 2483576 | 2483576 | 56,84 | 2483576 | 2483576 | 1 | 1 | 0 | 2098 |
|  | B. pseudocatenulatum DX_pv5_MiSeq | 14 | 534061 | 2458323 | 56,81 | 346328 | 301975 | 3 | 5 | 0 | 2096 |
|  | B. adolescentis PX1_F1pv2 | 14 | 1245413 | 2218233 | 59,56 | 1245413 | 173545 | 1 | 3 | 0 | 1812 |
|  | B. longum PX1_F1pv1 | 122 | 125472 | 2614853 | 60 | 57673 | 35161 | 16 | 31 | 0 | 2293 |
|  | B. longum PX1_F5pv1 | 72 | 220082 | 2437587 | 60,16 | 61767 | 35524 | 13 | 25 | 0 | 2080 |
|  | B. pseudocatenulatum PX1_F5pv2 | 15 | 865301 | 2475157 | 56,83 | 487475 | 162227 | 2 | 4 | 0 | 2118 |
|  | B. longum PX1_F7pv1 | 61 | 220546 | 2467116 | 60,19 | 86418 | 49537 | 10 | 20 | 0 | 2128 |
|  | B. longum PX1_F7pv2 | 114 | 125302 | 2528865 | 60,16 | 58486 | 33857 | 15 | 30 | 0 | 2217 |
|  | B. longum PX1_F7pv3 | 58 | 185850 | 2466891 | 60,2 | 87033 | 51531 | 10 | 20 | 0 | 2128 |
|  | B. longum PX1_F7pv4 | 108 | 125302 | 2525520 | 60,18 | 58484 | 37229 | 15 | 29 | 0 | 2206 |
|  | B. longum PX2_F1pv2 | 52 | 261002 | 2397423 | 60,13 | 118909 | 69181 | 7 | 13 | 0 | 2029 |
|  | B. pseudocatenulatum PX2_F1pv3 | 19 | 865113 | 2475212 | 56,84 | 441667 | 116946 | 2 | 5 | 0 | 2123 |
|  | B. animalis PX2_F5pv2 | 14 | 531306 | 1919033 | 60,46 | 439237 | 114331 | 2 | 5 | 0 | 1560 |
|  | B. longum PX2_F5pv5 | 46 | 297632 | 2481676 | 59,95 | 136423 | 92629 | 7 | 12 | 0 | 2112 |
|  | B. pseudocatenulatum PX2_F5pv1 | 18 | 856175 | 2458892 | 56,81 | 441078 | 145866 | 2 | 5 | 0 | 2095 |
|  | B. adolescentis PX3_F1pv3 | 15 | 1523158 | 2217947 | 59,56 | 1523158 | 174479 | 1 | 2 | 0 | 1809 |
|  | B. longum PX3_F1pv1 | 61 | 260094 | 2400487 | 60,11 | 118909 | 57433 | 7 | 14 | 0 | 2031 |
|  | B. longum PX3_F1pv2 | 115 | 230406 | 2613830 | 59,94 | 82926 | 37311 | 10 | 22 | 0 | 2336 |
|  | B. adolescentis PX3_F4pv3 | 14 | 1337467 | 2221401 | 59,56 | 1337467 | 173543 | 1 | 3 | 0 | 1814 |
|  | B. longum PX3_F4pv2 | 103 | 125302 | 2474362 | 60,22 | 63507 | 37229 | 15 | 29 | 0 | 2134 |
|  | B. longum PX3_F4pv4 | 46 | 293849 | 2531813 | 60,02 | 136467 | 106656 | 7 | 12 | 0 | 2149 |
|  | B. longum PX3_F4pv5 | 104 | 126661 | 2524007 | 60,21 | 70571 | 36133 | 14 | 27 | 0 | 2213 |
|  | B. pseudocatenulatum PX3_F4pv1 | 20 | 856145 | 2474538 | 56,84 | 441181 | 129458 | 2 | 5 | 0 | 2122 |
|  | B. adolescentis PX4_F2pv4 | 13 | 1523170 | 2218143 | 59,56 | 1523170 | 173545 | 1 | 2 | 0 | 1811 |
|  | B. bifidum PX4_F2pv2 | 13 | 476998 | 2246490 | 62,66 | 391211 | 213688 | 3 | 5 | 0 | 1920 |
|  | B. longum PX4_F2pv1 | 53 | 261106 | 2394152 | 60,13 | 111558 | 69181 | 7 | 13 | 0 | 2022 |
|  | B. longum PX4_F2pv3 | 108 | 125302 | 2699016 | 59,97 | 57674 | 34589 | 17 | 32 | 0 | 2415 |
|  | B. adolescentis PX4_F7pv4 | 14 | 1522848 | 2221780 | 59,56 | 1522848 | 173543 | 1 | 2 | 0 | 1814 |
|  | B. longum PX4_F7pv1 | 57 | 213869 | 2408363 | 60,12 | 88704 | 52309 | 9 | 17 | 0 | 2041 |
|  | B. longum PX4_F7pv2 | 44 | 255060 | 2373752 | 60,17 | 135623 | 76815 | 6 | 12 | 0 | 2000 |
|  | B. bifidum PX6_F1pv4 | 15 | 477005 | 2248466 | 62,66 | 309986 | 159073 | 3 | 6 | 0 | 1921 |
|  | B. longum PX6_F1pv1 | 72 | 237589 | 2593426 | 59,91 | 90377 | 41009 | 9 | 20 | 0 | 2280 |
|  | B. longum PX6_F1pv2 | 62 | 261311 | 2474123 | 60,2 | 106118 | 72215 | 8 | 15 | 0 | 2091 |
|  | B. longum PX6_F1pv3 | 108 | 125472 | 2493730 | 60,23 | 55445 | 36429 | 16 | 30 | 0 | 2192 |
|  | B. longum PX6_F7pv1 | 106 | 125472 | 2472559 | 60,23 | 63507 | 37729 | 15 | 28 | 0 | 2137 |
|  | B. longum PX6_F7pv2 | 53 | 242638 | 2397044 | 60,12 | 95023 | 64455 | 8 | 15 | 0 | 2029 |
|  | B. longum PX7_F1pv5 | 44 | 453156 | 2393856 | 60,18 | 118023 | 76814 | 6 | 12 | 0 | 2032 |
|  | B. longum PX7_F1pv6 | 97 | 124717 | 2652542 | 59,97 | 70469 | 37230 | 15 | 29 | 0 | 2341 |
|  | B. pseudocatenulatum PX7_F1pv7 | 18 | 865113 | 2433370 | 56,78 | 516886 | 239286 | 2 | 4 | 0 | 2059 |
|  | B. longum PX7_F2pv11 | 51 | 243155 | 2528972 | 60,02 | 155879 | 87242 | 7 | 12 | 0 | 2159 |
|  | B. pseudocatenulatum PX7_F2pv8 | 21 | 865109 | 2475733 | 56,83 | 441378 | 129458 | 2 | 5 | 0 | 2125 |
|  | B. longum PX7_F7pv1 | 56 | 261057 | 2427240 | 59,89 | 146984 | 57428 | 7 | 13 | 0 | 2053 |
|  | B. longum PX7_F7pv2 | 43 | 385880 | 2433906 | 59,87 | 146984 | 92629 | 6 | 11 | 0 | 2055 |
|  | B. longum DY_pv2 | 59 | 232709 | 2422758 | 60,33 | 91460 | 52699 | 9 | 18 | 0 | 2067 |
|  | B. longum DY_pv11 | 31 | 388830 | 2425560 | 60,32 | 130083 | 90927 | 6 | 11 | 0 | 2057 |
|  | B. pseudocatenulatum DY_pv1 | 48 | 576145 | 2400471 | 56,28 | 233658 | 137515 | 3 | 7 | 0 | 2019 |
|  | B. longum PY1_F4pv2 | 36 | 357486 | 2427435 | 60,33 | 127759 | 86215 | 7 | 13 | 0 | 2069 |
|  | B. longum PY1_F7pv1 | 35 | 357488 | 2386851 | 60,32 | 130081 | 90927 | 7 | 12 | 0 | 2004 |
|  | B. longum PY5_F1pv2 | 31 | 478199 | 2426210 | 60,32 | 131697 | 104727 | 5 | 10 | 0 | 2056 |
|  | B. longum PY5_F7pv1 | 38 | 356774 | 2372202 | 60,32 | 130082 | 79476 | 7 | 12 | 0 | 2004 |
|  | B. longum PY5_F7pv4 | 29 | 437421 | 2371681 | 60,32 | 133442 | 89299 | 5 | 10 | 0 | 1999 |

**Supplementary Table 5. Summary of comparative genomics.**

| **Isolate** | **rep-PCR type** | **Pangenome analysis^1^** | **Phylogenomic analysis^2^** | **Phylogenetic analysis^3^** | **Genome assembly BLAST hit^4^** |
| --- | --- | --- | --- | --- | --- |
| B. adolescentis DX_pv1 | DX-1 | DX-1 group | DX-1 group | DX-1 group | Bifidobacterium adolescentis ATCC 15703 DNA, complete genome; NC_008618.1 |
| B. adolescentis DX_pv54 | DX-1-like | DX-1 group | DX-1 group | DX-1 group | Bifidobacterium adolescentis ATCC 15703 DNA, complete genome; NC_008618.1 |
| B. animalis DX_pv4 | DX-4 | B. pseudolongum group | B. pseudolongum group | not done | Bifidobacterium pseudolongum PV8-2, complete genome; NZ_CP007457.1 |
| B. bifidum DX_pv3 | DX-3 | B. bifidum group | B. bifidum group | not done | Bifidobacterium bifidum PRL2010 chromosome, complete genome; NC_014638.1 |
| B. longum DX_pv2 | DX-2 | DX-2 group | DX-2 group | DX-2 group | Bifidobacterium longum NCC2705 chromosome, complete genome; NC_004307.2 |
| B. longum DX_pv7 | DX-2 | DX-2 group | DX-7 group | DX-2 group | Bifidobacterium longum NCC2705 chromosome, complete genome; NC_004307.2 |
| B. longum DX_pv16 | DX-2-like | DX-2 group | DX-7 group | DX-2 group | Bifidobacterium longum NCC2705 chromosome, complete genome; NC_004307.2 |
| B. longum DX_pv18 | DX-18 | DX-18 group | DX-18 group | DX-18 group | Bifidobacterium longum NCC2705 chromosome, complete genome; NC_004307.2 |
| B. longum DX_pv23 | DX-23 | DX-23 group | DX-23 group | DX-23 group | Bifidobacterium longum NCC2705 chromosome, complete genome; NC_004307.2 |
| B. longum DX_pv32_PacBio | DX-2-like | DX-2 group | alone | DX-2 group | contigs 0, 1, 2, 3, and 5: Bifidobacterium longum NCC2705 chromosome, complete genome; NC_004307.2; contig 4: Bifidobacterium longum NCC2705 plasmid pBLO1, complete sequence; NC_004943.1 |
| B. longum DX_pv32_MiSeq | DX-2-like | DX-2 group | DX-2 group | DX-2 group | Bifidobacterium longum NCC2705 chromosome, complete genome; NC_004307.2 |
| B. longum DX_pv36 | DX-18-like | DX-18 group | DX-18 group | DX-18 group | Bifidobacterium longum NCC2705 chromosome, complete genome; NC_004307.2 |
| B. longum DX_pv48 | DX-23-like | DX-23 group | DX-23 group | DX-23 group | Bifidobacterium longum NCC2705 chromosome, complete genome; NC_004307.2 |
| B. longum DX_pv51 | DX-2-like | DX-2 group | DX-7 group | not done | Bifidobacterium longum strain 51A chromosome, complete genome; NZ_CP026999.1 |
| B. longum DX_pv52 | DX-2-like | DX-2 group | DX-7 group | DX-2 group | Bifidobacterium longum NCC2705 chromosome, complete genome; NC_004307.2 |
| B. longum DX_pv53 | DX-23-like | DX-23 group | DX-23 group | DX-23 group | Bifidobacterium longum NCC2705 chromosome, complete genome; NC_004307.2 |
| B. pseudocatenulatum DX_pv5_PacBio | DX-5 | DX-5 group | DX-5 group | DX-5 group | Bifidobacterium pseudocatenulatum DSM 20438 = JCM 1200 = LMG 10505 DNA, complete genome; AP012330.1 |
| B. pseudocatenulatum DX_pv5_MiSeq | DX-5 | DX-5 group | DX-5 group | DX-5 group | Bifidobacterium pseudocatenulatum DSM 20438 = JCM 1200 = LMG 10505 DNA, complete genome; AP012330.1 |
| B. adolescentis PX1_F1pv2 | PX1-F1-2 | DX-1 group | DX-1 group | DX-1 group | not done |
| B. longum PX1_F1pv1 | DX-18-like | DX-18 group | DX-18 group | DX-18 group | not done |
| B. longum PX1_F5pv1 | DX-2-like | DX-2 group | alone | DX-2 group | not done |
| B. pseudocatenulatum PX1_F5pv2 | DX-5-like | DX-5 group | DX-5 group | DX-5 group | not done |
| B. longum PX1_F7pv1 | DX-2-like | DX-2 group | DX-18 group | DX-2 group | not done |
| B. longum PX1_F7pv2 | DX-18-like | DX-18 group | DX-18 group | DX-18 group | not done |
| B. longum PX1_F7pv3 | DX-2-like | DX-2 group | DX-18 group | DX-2 group | not done |
| B. longum PX1_F7pv4 | DX-18-like | DX-18 group | DX-18 group | DX-18 group | not done |
| B. longum PX2_F1pv2 | DX-23-like | DX-23 group | DX-23 group | DX-23 group | not done |
| B. pseudocatenulatum PX2_F1pv3 | DX-5-like | DX-5 group | DX-5 group | DX-5 group | not done |
| B. animalis PX2_F5pv2 | DX-4-like | B. pseudolongum group | B. pseudolongum group | not done | not done |
| B. longum PX2_F5pv5 | PX2-F5-5 | PX group | PX group | PX group | not done |
| B. pseudocatenulatum PX2_F5pv1 | DX-5-like | DX-5 group | DX-5 group | DX-5 group | not done |
| Bifidobacterium sp. PX3_F1pv3 | DX-1-like | DX-1 group | DX-1 group | DX-1 group | not done |
| B. longum PX3_F1pv1 | DX-23-like | DX-23 group | DX-23 group | DX-23 group | not done |
| B. longum PX3_F1pv2 | DX-2-like | DX-2 group | DX-2 group | DX-2 group | not done |
| B. adolescentis PX3_F4pv3 | DX-1-like | DX-1 group | DX-1 group | DX-1 group | not done |
| B. longum PX3_F4pv2 | DX-18-like | DX-18 group | DX-18 group | DX-18 group | not done |
| B. longum PX3_F4pv4 | PX3-F4-4 | PX group | PX group | PX group | not done |
| B. longum PX3_F4pv5 | DX-18-like | DX-18 group | DX-18 group | DX-18 group | not done |
| B. pseudocatenulatum PX3_F4pv1 | DX-5-like | DX-5 group | DX-5 group | DX-5 group | not done |
| B. adolescentis PX4_F2pv4 | DX-1-like | DX-1 group | DX-1 group | DX-1 group | not done |
| B. bifidum PX4_F2pv2 | DX-3-like | B. bifidum group | B. bifidum group | not done | not done |
| B. longum PX4_F2pv1 | DX-23-like | DX-23 group | DX-23 group | DX-23 group | not done |
| B. longum PX4_F2pv3 | DX-18-like | DX-18 group | DX-18 group | DX-18 group | not done |
| B. adolescentis PX4_F7pv4 | DX-1-like | DX-1 group | DX-1 group | DX-1 group | not done |
| B. longum PX4_F7pv1 | DX-23-like | DX-23 group | DX-23 group | DX-23 group | not done |
| B. longum PX4_F7pv2 | DX-23-like | DX-23 group | alone | DX-23 group | not done |
| B. bifidum PX6_F1pv4 | DX-3-like | B. bifidum group | B. bifidum group | not done | not done |
| B. longum PX6_F1pv1 | DX-2-like | DX-2 group | DX-2 group | DX-2 group | not done |
| B. longum PX6_F1pv2 | DX-23-like | DX-23 group | DX-7 group | DX-23 group | not done |
| B. longum PX6_F1pv3 | DX-18-like | DX-18 group | DX-18 group | DX-18 group | not done |
| B. longum PX6_F7pv1 | DX-18-like | DX-18 group | DX-18 group | DX-18 group | not done |
| B. longum PX6_F7pv2 | DX-23-like | DX-23 group | DX-23 group | DX-23 group | not done |
| B. longum PX7_F1pv5 | DX-23-like | DX-23 group | alone | DX-23 group | not done |
| B. longum PX7_F1pv6 | DX-18-like | DX-18 group | DX-18 group | DX-18 group | not done |
| B. pseudocatenulatum PX7_F1pv7 | DX-5-like | DX-5 group | DX-5 group | DX-5 group | not done |
| B. longum PX7_F2pv11 | PX7-F2-11 | PX group | PX group | PX group | not done |
| B. pseudocatenulatum PX7_F2pv8 | DX-5-like | DX-5 group | DX-5 group | DX-5 group | not done |
| B. longum PX7_F7pv1 | PX7-F2-11-like | PX group | PX group | PX group | not done |
| B. longum PX7_F7pv2 | PX7-F2-11-like | PX group | PX group | PX group | not done |
| B. longum DY_pv2 | DY-2 | DY-2 group | DY-2 group | DY-2 group | Bifidobacterium longum NCC2705 chromosome, complete genome; NC_004307.2 |
| B. longum DY_pv11 | DY-2-like | DY-2 group | DY-2 group | DY-2 group | Bifidobacterium longum NCC2705 chromosome, complete genome; NC_004307.2 |
| B. pseudocatenulatum DY_pv1 | DY-1 | alone | alone | alone | Bifidobacterium pseudocatenulatum strain CECT 7765, whole genome shotgun sequence; NZ_CDPW01000001.1 |
| B. longum PY1_F4pv2 | DY-2-like | DY-2 group | alone | not done | not done |
| B. longum PY1_F7pv1 | DY-2-like | DY-2 group | DY-2 group | DY-2 group | not done |
| B. longum PY5_F1pv2 | DY-2-like | DY-2 group | DY-2 group | DY-2 group | not done |
| B. longum PY5_F7pv1 | DY-2-like | DY-2 group | alone | DY-2 group | not done |
| B. longum PY5_F7pv4 | DY-2-like | DY-2 group | alone | DY-2 group | not done |

^1^Comparison and visualization of gene clusters among the genomes.

^2^Phylogenomic analysis of 49 concatenated ribosomal proteins.

^3^Phylogenetic analysis of whole genome SNPs.

^4^The best BLAST hit (i.e. sequence producing most significant alignment) from the NCBI RefSeq genome database for a single-line fasta-file of genome assembly; NCBI Reference Sequence

**Supplementary Table 6. SNP matrices from CSI Phylogeny tool.**

| **B. longum DX-18 group+B3:N62** | DX_ pv36 | PX4_ F2pv3 | PX7_ F1pv6 | PX1_ F7pv2 | PX1_ F7pv4 | PX3_ F4pv5 | PX1_ F1pv1 | PX6_ F7pv1 | PX3_ F4pv2 | DX_ pv18 | PX6_ F1pv3 | B_longum refseq NC_004307.2 | B_longum refseq NZ_CP026999.1 |
| --- | --- | --- | --- | --- | --- | --- | --- | --- | --- | --- | --- | --- | --- |
| DX_pv36 | 0 | 5 | 22 | 165 | 187 | 401 | 406 | 406 | 427 | 496 | 841 | 7509 | 7701 |
| PX4_F2pv3 | 5 | 0 | 19 | 162 | 184 | 398 | 403 | 401 | 424 | 493 | 838 | 7508 | 7696 |
| PX7_F1pv6 | 22 | 19 | 0 | 179 | 167 | 415 | 420 | 420 | 441 | 510 | 855 | 7511 | 7703 |
| PX1_F7pv2 | 165 | 162 | 179 | 0 | 26 | 256 | 359 | 359 | 376 | 571 | 808 | 7588 | 7742 |
| PX1_F7pv4 | 187 | 184 | 167 | 26 | 0 | 278 | 381 | 381 | 398 | 593 | 830 | 7588 | 7748 |
| PX3_F4pv5 | 401 | 398 | 415 | 256 | 278 | 0 | 107 | 107 | 124 | 319 | 556 | 7586 | 7720 |
| PX1_F1pv1 | 406 | 403 | 420 | 359 | 381 | 107 | 0 | 4 | 47 | 324 | 651 | 7543 | 7709 |
| PX6_F7pv1 | 406 | 401 | 420 | 359 | 381 | 107 | 4 | 0 | 47 | 324 | 651 | 7541 | 7707 |
| PX3_F4pv2 | 427 | 424 | 441 | 376 | 398 | 124 | 47 | 47 | 0 | 345 | 674 | 7538 | 7684 |
| DX_pv18 | 496 | 493 | 510 | 571 | 593 | 319 | 324 | 324 | 345 | 0 | 671 | 7493 | 7683 |
| PX6_F1pv3 | 841 | 838 | 855 | 808 | 830 | 556 | 651 | 651 | 674 | 671 | 0 | 7533 | 7694 |
| B_longum refseq NC_004307.2 | 7509 | 7508 | 7511 | 7588 | 7588 | 7586 | 7543 | 7541 | 7538 | 7493 | 7533 | 0 | 7562 |
| B_longum refseq NZ_CP026999.1 | 7701 | 7696 | 7703 | 7742 | 7748 | 7720 | 7709 | 7707 | 7684 | 7683 | 7694 | 7562 | 0 |
| **B. longum DX-2 group** | DX_ pv16 | DX_ pv32 | DX_ pv2 | DX_ pv51 | DX_ pv52 | DX_ pv7 | PX1_ F5pv1 | PX1_ F7pv1 | PX1_ F7pv3 | PX3_ F1pv2 | PX6_ F1pv1 | B_longum refseq NC_004307.2 | B_longum refseq NZ_CP026999.1 |
| DX_pv16 | 0 | 1128 | 1048 | 1981 | 1983 | 2083 | 1206 | 2130 | 2131 | 1204 | 1706 | 7268 | 7293 |
| DX_pv32 | 1128 | 0 | 322 | 1487 | 1489 | 1609 | 1018 | 1885 | 1886 | 482 | 1012 | 7291 | 7309 |
| DX_pv2 | 1048 | 322 | 0 | 1173 | 1175 | 1295 | 938 | 1687 | 1688 | 172 | 698 | 7289 | 7355 |
| DX_pv51 | 1981 | 1487 | 1173 | 0 | 6 | 630 | 2105 | 2356 | 2357 | 1147 | 581 | 7217 | 7258 |
| DX_pv52 | 1983 | 1489 | 1175 | 6 | 0 | 632 | 2107 | 2358 | 2359 | 1149 | 583 | 7219 | 7260 |
| DX_pv7 | 2083 | 1609 | 1295 | 630 | 632 | 0 | 2225 | 2616 | 2617 | 1243 | 733 | 7190 | 7192 |
| PX1_F5pv1 | 1206 | 1018 | 938 | 2105 | 2107 | 2225 | 0 | 1126 | 1127 | 1098 | 1594 | 7411 | 7479 |
| PX1_F7pv1 | 2130 | 1885 | 1687 | 2356 | 2358 | 2616 | 1126 | 0 | 3 | 1705 | 2049 | 7477 | 7520 |
| PX1_F7pv3 | 2131 | 1886 | 1688 | 2357 | 2359 | 2617 | 1127 | 3 | 0 | 1706 | 2050 | 7478 | 7521 |
| PX3_F1pv2 | 1204 | 482 | 172 | 1147 | 1149 | 1243 | 1098 | 1705 | 1706 | 0 | 698 | 7309 | 7399 |
| PX6_F1pv1 | 1706 | 1012 | 698 | 581 | 583 | 733 | 1594 | 2049 | 2050 | 698 | 0 | 7201 | 7167 |
| B_longum refseq NC_004307.2 | 7268 | 7291 | 7289 | 7217 | 7219 | 7190 | 7411 | 7477 | 7478 | 7309 | 7201 | 0 | 7562 |
| B_longum refseq NZ_CP026999.1 | 7293 | 7309 | 7355 | 7258 | 7260 | 7192 | 7479 | 7520 | 7521 | 7399 | 7167 | 7562 | 0 |
| **B. longum DX-23 group** | DX_ pv23 | DX_ pv48 | DX_ pv53 | PX2_ F1pv2 | PX3_ F1pv1 | PX4_ F2pv1 | PX6_ F7pv2 | PX4_ F7pv1 | PX4_ F7pv2 | PX7_ F1pv5 | PX6_ F1pv2 | B_longum refseq NC_004307.2 | B_longum refseq NZ_CP026999.1 |
| DX_pv23 | 0 | 2 | 5 | 4 | 6 | 6 | 3 | 993 | 2389 | 2389 | 2152 | 7234 | 7566 |
| DX_pv48 | 2 | 0 | 5 | 4 | 6 | 6 | 3 | 993 | 2389 | 2389 | 2152 | 7234 | 7566 |
| DX_pv53 | 5 | 5 | 0 | 7 | 9 | 9 | 6 | 992 | 2392 | 2392 | 2153 | 7235 | 7565 |
| PX2_F1pv2 | 4 | 4 | 7 | 0 | 8 | 8 | 5 | 995 | 2391 | 2391 | 2154 | 7236 | 7568 |
| PX3_F1pv1 | 6 | 6 | 9 | 8 | 0 | 10 | 7 | 997 | 2393 | 2393 | 2156 | 7230 | 7570 |
| PX4_F2pv1 | 6 | 6 | 9 | 8 | 10 | 0 | 5 | 997 | 2391 | 2391 | 2156 | 7236 | 7568 |
| PX6_F7pv2 | 3 | 3 | 6 | 5 | 7 | 5 | 0 | 994 | 2388 | 2390 | 2153 | 7235 | 7567 |
| PX4_F7pv1 | 993 | 993 | 992 | 995 | 997 | 997 | 994 | 0 | 3276 | 3276 | 2232 | 7249 | 7625 |
| PX4_F7pv2 | 2389 | 2389 | 2392 | 2391 | 2393 | 2391 | 2388 | 3276 | 0 | 13 | 3731 | 7426 | 7486 |
| PX7_F1pv5 | 2389 | 2389 | 2392 | 2391 | 2393 | 2391 | 2390 | 3276 | 13 | 0 | 3731 | 7425 | 7484 |
| PX6_F1pv2 | 2152 | 2152 | 2153 | 2154 | 2156 | 2156 | 2153 | 2232 | 3731 | 3731 | 0 | 7165 | 7433 |
| B_longum refseq NC_004307.2 | 7234 | 7234 | 7235 | 7236 | 7230 | 7236 | 7235 | 7249 | 7426 | 7425 | 7165 | 0 | 7562 |
| B_longum refseq NZ_CP026999.1 | 7566 | 7566 | 7565 | 7568 | 7570 | 7568 | 7567 | 7625 | 7486 | 7484 | 7433 | 7562 | 0 |
| **B. longum PX-group** | PX2_ F5pv5 | PX3_ F4pv4 | PX7_ F2pv11 | PX7_ F7pv1 | PX7_ F7pv2 | DX_ pv18 | DX_ pv23 | DX_ pv2 | DY_ pv2 |  |  | B_longum refseq NC_004307.2 | B_longum refseq NZ_CP026999.1 |
| PX2_F5pv5 | 0 | 3 | 4 | 11 | 11 | 7062 | 6691 | 6569 | 7810 |  |  | 7298 | 7637 |
| PX3_F4pv4 | 3 | 0 | 3 | 10 | 10 | 7061 | 6690 | 6568 | 7809 |  |  | 7297 | 7636 |
| PX7_F2pv11 | 4 | 3 | 0 | 11 | 11 | 7062 | 6691 | 6569 | 7810 |  |  | 7298 | 7637 |
| PX7_F7pv1 | 11 | 10 | 11 | 0 | 14 | 7069 | 6698 | 6576 | 7817 |  |  | 7305 | 7644 |
| PX7_F7pv2 | 11 | 10 | 11 | 14 | 0 | 7067 | 6698 | 6572 | 7817 |  |  | 7305 | 7642 |
| DX_pv18 | 7062 | 7061 | 7062 | 7069 | 7067 | 0 | 6157 | 5232 | 8109 |  |  | 7493 | 7683 |
| DX_pv23 | 6691 | 6690 | 6691 | 6698 | 6698 | 6157 | 0 | 3765 | 8172 |  |  | 7234 | 7566 |
| DX_pv2 | 6569 | 6568 | 6569 | 6576 | 6572 | 5232 | 3765 | 0 | 8045 |  |  | 7289 | 7355 |
| DY_pv2 | 7810 | 7809 | 7810 | 7817 | 7817 | 8109 | 8172 | 8045 | 0 |  |  | 7817 | 7692 |
| B_longum refseq NC_004307.2 | 7298 | 7297 | 7298 | 7305 | 7305 | 7493 | 7234 | 7289 | 7817 |  |  | 0 | 7562 |
| B_longum refseq NZ_CP026999.1 | 7637 | 7636 | 7637 | 7644 | 7642 | 7683 | 7566 | 7355 | 7692 |  |  | 7562 | 0 |
| **B. longum DY-2 group** | DY_ pv2 | DY_ pv11 | PY5_ F1pv2 | PY1_ F4pv2 | PY1_ F7pv1 | PY5_ F7pv1 | PY5_ F7pv4 |  |  |  |  | B_longum refseq NC_004307.2 | B_longum refseq NZ_CP026999.1 |
| DY_pv2 | 0 | 182 | 189 | 245 | 218 | 1198 | 1234 |  |  |  |  | 7817 | 7692 |
| DY_pv11 | 182 | 0 | 11 | 289 | 262 | 1016 | 1056 |  |  |  |  | 7879 | 7730 |
| PY5_F1pv2 | 189 | 11 | 0 | 296 | 269 | 1023 | 1063 |  |  |  |  | 7884 | 7735 |
| PY1_F4pv2 | 245 | 289 | 296 | 0 | 51 | 1193 | 1277 |  |  |  |  | 7786 | 7659 |
| PY1_F7pv1 | 218 | 262 | 269 | 51 | 0 | 1224 | 1308 |  |  |  |  | 7797 | 7678 |
| PY5_F7pv1 | 1198 | 1016 | 1023 | 1193 | 1224 | 0 | 558 |  |  |  |  | 7658 | 7781 |
| PY5_F7pv4 | 1234 | 1056 | 1063 | 1277 | 1308 | 558 | 0 |  |  |  |  | 7789 | 7799 |
| B_longum refseq NC_004307.2 | 7817 | 7879 | 7884 | 7786 | 7797 | 7658 | 7789 |  |  |  |  | 0 | 7562 |
| B_longum refseq NZ_CP026999.1 | 7692 | 7730 | 7735 | 7659 | 7678 | 7781 | 7799 |  |  |  |  | 7562 | 0 |
| **B. adolescentis DX-1 group** | DX_ pv1 | DX_ pv54 | PX1_ F1pv2 | PX3_ F1pv3 | PX3_ F4pv3 | PX4_ F2pv4 | PX4_ F7pv4 |  | |  |  | B_adolescentis refseq NC_008618.1 |  |
| DX_pv1 | 0 | 439 | 436 | 5 | 524 | 435 | 526 |  |  |  |  | 8413 |  |
| DX_pv54 | 439 | 0 | 17 | 442 | 105 | 16 | 107 |  |  |  |  | 8420 |  |
| PX1_F1pv2 | 436 | 17 | 0 | 439 | 102 | 3 | 104 |  |  |  |  | 8417 |  |
| PX3_F1pv3 | 5 | 442 | 439 | 0 | 527 | 438 | 529 |  |  |  |  | 8416 |  |
| PX3_F4pv3 | 524 | 105 | 102 | 527 | 0 | 101 | 4 |  |  |  |  | 8405 |  |
| PX4_F2pv4 | 435 | 16 | 3 | 438 | 101 | 0 | 103 |  |  |  |  | 8416 |  |
| PX4_F7pv4/ | 526 | 107 | 104 | 529 | 4 | 103 | 0 |  |  |  |  | 8407 |  |
| B_adolescentis refseq NC_008618.1 | 8413 | 8420 | 8417 | 8416 | 8405 | 8416 | 8407 |  |  |  |  | 0 |  |
| **B. pseudocatenulatum DX-5 group** | DX_ pv5 MiSeq | PX2_ F1pv3 | PX2_ F5pv1 | PX7_ F1pv7 | PX7_ F2pv8 | PX3_ F4pv1 | PX1_ F5pv2 | DY_ pv1 |  | |  | B_pseudocatenulatum refseq AP012330.1 |  |
| DX_pv5 | 0 | 5 | 8 | 4 | 4 | 129 | 129 | 7950 |  |  |  | 6767 |  |
| PX2_F1pv3 | 5 | 0 | 11 | 7 | 5 | 130 | 130 | 7951 |  |  |  | 6768 |  |
| PX2_F5pv1 | 8 | 11 | 0 | 10 | 8 | 131 | 133 | 7954 |  |  |  | 6771 |  |
| PX7_F1pv7 | 4 | 7 | 10 | 0 | 6 | 131 | 131 | 7952 |  |  |  | 6769 |  |
| PX7_F2pv8 | 4 | 5 | 8 | 6 | 0 | 127 | 129 | 7950 |  |  |  | 6767 |  |
| PX3_F4pv1 | 129 | 130 | 131 | 131 | 127 | 0 | 10 | 7857 |  |  |  | 6697 |  |
| PX1_F5pv2 | 129 | 130 | 133 | 131 | 129 | 10 | 0 | 7855 |  |  |  | 6695 |  |
| DY_pv1 | 7950 | 7951 | 7954 | 7952 | 7950 | 7857 | 7855 | 0 |  |  |  | 8156 |  |
| B_pseudocatenulatum refseq AP012330.1 | 6767 | 6768 | 6771 | 6769 | 6767 | 6697 | 6695 | 8156 |  |  |  | 0 |  |

**Supplementary Figure 1**. Clustering of *Bifidobacterium*-specific PCR-DGGE profiles obtained from fecal samples of FMT donors and their recipients at different time points. Dendrogram presents all the samples after UPGMA cluster analysis using Pearson similarity coefficient.

DGGE = denaturing gradient gel electrophoresis. UPGMA = unweighted pair group method with arithmetic mean. DX and DY = FMT donors. PX1-7 and PY1-6 = FMT recipients of DX and DY, respectively. Fecal sample time points for recipients and DY: F0 = pre-FMT (the time of donation for DY), F1-F7 = 3 days, 2 weeks, 1, 2, 4, 8, and 12 months post-FMT, respectively. F0n = PCR product obtained from nested PCR. Time points for DX: F3 = the time of donation, F4, F5, F7, F10, F13, and F14 = 2 weeks, 1, 2, 4, 8, and 12 months post-FMT, respectively.


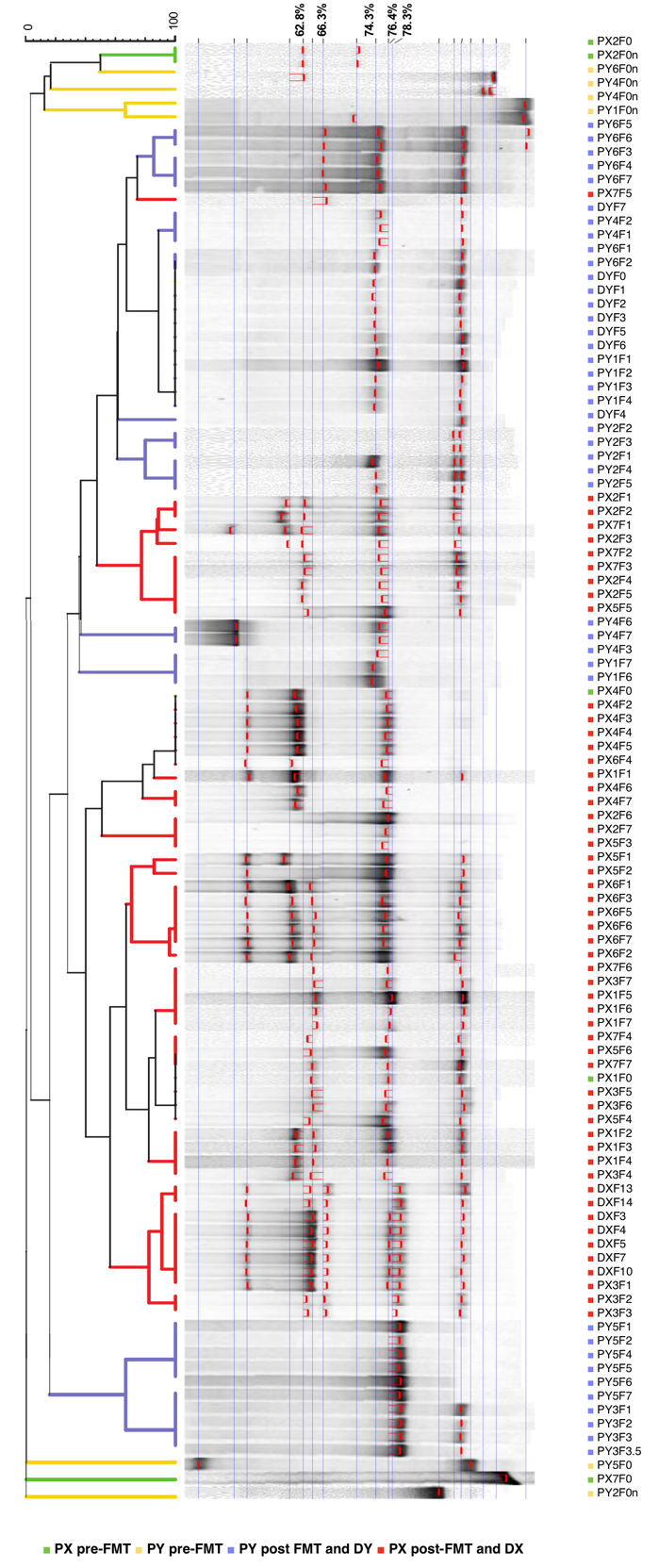


**Supplementary Figure 2.** Phylogenetic trees of fecal bifidobacterial isolates from FMT donors and their recipients. a) Phylogenetic tree of *B. pseudocatenulatum* isolates based on whole genome SNP calling.

b) Phylogenetic tree of *B. adolescentis* isolates based on whole genome SNP calling.

DX and DY = FMT donors. PX1-4 and PX6-7 = FMT recipients of DX, PY1 and PY5 = FMT recipients of DY. F1, F2, F4, F5, and F7 = 3 days, 2 weeks, 2, 4, and 12 months post-FMT, respectively. pv1-54 = isolate codes. DX_pv5_PacBio and DX_pv32_PacBio = two DX isolate genomes that were sequenced by PacBio in addition to MiSeq. REF = reference strains from the NCBI reference genome database (Supplementary Table 5).


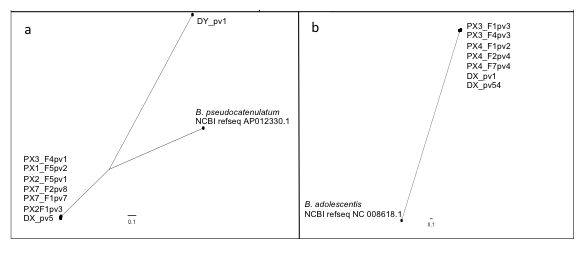

Supplement: Supplementary file 1 [file Data_Sheet_1.docx]
